# Effect of Chitosan and Alginate-Based Edible Membranes with Oregano Essential Oil and Olive Oil in the Microbiological, Physicochemical and Organoleptic Characteristics of Mutton

**DOI:** 10.3390/microorganisms11020507

**Published:** 2023-02-17

**Authors:** Anestis Tsitsos, Vangelis Economou, Eirini Chouliara, Georgia Koutouzidou, Georgios Arsenos, Ioannis Ambrosiadis

**Affiliations:** 1 Laboratory of Animal Food Products Hygiene and Veterinary Public Health, School of Veterinary Medicine, Aristotle University of Thessaloniki, 54124 Thessaloniki, Greece; 2 Laboratory of Technology of Food Animal Origin, School of Veterinary Medicine, Aristotle University of Thessaloniki, 54124 Thessaloniki, Greece; 3 Department of Agriculture, School of Agricultural Sciences, University of Western Macedonia, 53100 Florina, Greece; 4 Laboratory of Animal Husbandry, School of Veterinary Medicine, Aristotle University of Thessaloniki, 54124 Thessaloniki, Greece

**Keywords:** mutton, sheep, chitosan, alginates, oregano essential oil, vacuum, edible membranes, microbiological quality, colour

## Abstract

Edible chitosan or alginate coatings and their combinations with oregano essential oil or olive oil, have been examined for their effect on the microbiological, physicochemical and organoleptic characteristics of mutton. The results indicated that these edible coatings can contribute to maintaining good quality characteristics and extending mutton shelf-life. The total mesophilic counts in mutton ranged from 3.48 to 8.00 log^10^ CFU/g, the total psychrophilic counts from 4.00 to 9.50 log^10^ CFU/g, the *B. thermosphacta* counts from 2.30 to 7.77 log^10^ CFU/g and the lactic acid bacteria counts from 2.00 to 5.85 log^10^ CFU/g. Chitosan coatings significantly (*p* < 0.05) reduced the total mesophilic, the total psychrophilic (1–2 log_10_ cfu/g), the *B. thermosphacta* and the lactic acid bacteria counts in mutton. Alginate exhibited a lower L* value and a higher a* value and chroma compared with the control and chitosan lots. No significant differences were observed in the chemical composition of meat pieces among the experimental groups. Oregano oil positively affected the sensory attributes of meat. The most favourable combination, based on the microbiological counts, the organoleptic characteristics and the shelf-life extension of mutton, was that of chitosan with oregano essential oil.

## 1. Introduction

Sheep meat is one of the most consumed types of meat worldwide [1,2]. It is considered an essential part of the human diet, as it provides a wide range of essential nutrients. Sheep meat is an excellent source of proteins with high biological value, essential amino acids, vitamin B complex (vitamin B_6,_ vitamin B_12_, pyridoxine, niacin, riboflavin, pantothenic acid), minerals (iron, zinc, selenium, phosphorus) and long-chain omega-3 polyunsaturated fatty acids (PUFAs) [3,4]. However, meat is susceptible to bacterial contamination and degradation. To preserve quality, many thermal (i.e., cooking, smoking or dehydration) and non-thermal preservation methods (i.e., vacuum packaging, high hydrostatic pressure and edible coatings) are used in the meat industry [5]. Edible coatings are thin membranes that are applied to a wide variety of food products to improve their quality and to enhance their shelf life; chitosan and alginate coatings are substances that have potential applications in the meat industry [6].

Chitosan is a cationic polymer of N-acetyl-D-glucosamine and D-glucosamine units and is produced after the partial deacetylation of chitin [7]. It is generally recognized as a safe (GRAS) food additive and food preservative of natural origin by the Food and Drug Administration (FDA) of the United States [8]. Chitosan coatings are mainly used in the packaging of meat products as they form strong coatings and positively contribute to food quality [9]. Chitosan has distinctive antimicrobial properties; it is effective against Gram-positive, Gram-negative bacteria and fungi [10]. Sodium alginate is a natural anionic polysaccharide that is isolated from the cell wall of brown algae (*Phaeophyceae* spp.) [11]. It is composed of (1,4)-linked β-D-mannuronate and α-L-guluronate residues and can be cross-linked by the addition of divalent ions, such as Ca^2+^, to form strong coatings [12]. It is used as a stabilizer, thickener and gelling agent in many types of food, such as sauces, desserts and beverages [13]. Sodium alginate improves food quality by limiting oxygen interaction, enhancing water barrier qualities and preserving food flavour [12]. It is also biodegradable, biocompatible and can be used as a carrier of natural ingredients [14,15]. The FDA has recognized sodium alginate as a GRAS substance and the European Food Safety Authority has approved the use of alginate and related salts in defined amounts [11].

Food industries often combine edible membranes with essential oils or vegetable oils for a further improvement of food quality. Essential oils and vegetable oils are natural substances that are extracted from plants or vegetables, respectively, and possess numerous technological properties [12]. Oregano oil is an essential oil obtained from the plant *Origanum vulgare*. It has antimicrobial and antioxidant characteristics due to its two major components, namely carvacrol and thymol [15,16,17]. It is generally regarded as safe (GRAS) by the FDA, whereas a scientific opinion issued by EFSA Panel on Food Additives and Nutrient Sources has proposed a dietary intake of up to 2.0 mg/kg bw/day for women and 2.3 mg/kg bw/day for men, stating though that more data are needed [18]. Due to the lipophilic character of oregano essential oil, its use is limited by its hydrophobicity. In order to overcome this issue, several authors report on the application of stabilizing procedures, such as the incorporation in emulsions, liposomes and capsules, that can also enhance the bioactive effect of oregano essential oil [17,19,20]. Olive oil is an example of a vegetable oil extracted by olives (*Olea europaea*) that possesses antioxidant activities [16,21]. It is also of high nutritional value, due to its high concentration of healthy fatty acids and especially oleic acid [22,23]. It is also considered as GRAS by the FDA [11]. Since both oils tend to impart a strong flavour, they are often used in smaller amounts as embedded in edible coatings, an addition that improves food quality and safety [20,24].

The antimicrobial and technological properties of chitosan and alginate membranes, as well as their emulsions with essential oils or vegetable oils, have been well demonstrated in many food systems, such as bakery products, fruits, vegetables, beef, pork, fish and chicken meat [5,9]. However, there is scarce information about the application of these edible membranes in mutton and their impact on meat quality. Thus, the scope of this study was to investigate the effect of chitosan or alginate edible coatings, as well as their combinations with oregano essential oil or olive oil, on the microbiological, physicochemical and organoleptic characteristics of mutton.

## 2. Materials and Methods

### 2.1. Meat Samples

Mutton from the thigh (*M. quadriceps fermoris*, *M. biceps fermoris*, *M. semimembranosus* and *M. semitendinosus*) and shoulder (*M. brachialisi*, *M. triceps brachii* and *M. deltoideus*) was provided by a collaborating meat company, located in Northern Greece. The meat, which was produced locally by sheep slaughtered in the company’s abattoir, was transferred in insulated polystyrene boxes with ice to the Laboratory of Animal Food Products Hygiene and Veterinary Public Health, School of Veterinary Medicine, within two hours for analysis. To simulate the actual weight of the commercial meat packaging usually marketed at retail, pieces of approximately 200 g each were aseptically produced and stored under refrigeration before the application of edible coatings. For every lot, at least 18 mutton pieces were prepared accordingly, to sample three pieces in each different time of storage.

### 2.2. Preparation of Edible Coatings and Essential Oil Solutions

Five stocks of edible coating solutions and their emulsions with essential oils were produced. Chitosan solution was made at a final concentration of 1% by dissolving 1 g of chitosan of medium molecular weight obtained from crab shells (48165, Sigma Aldrich, St. Louis, MO, USA) in 100 mL of 1% glacial acetic acid (100056, Merck KGaA, Darmstadt, Germany) and stirring overnight at room temperature. Alginate solution was produced at a final concentration of 1.5% by dissolving 1.5 g of sodium alginate obtained from brown algae (alginic acid sodium salt BioChemica, A3249, AppliChem GmBh, Darmstadt, Germany) in 100 mL of deionized water and stirring overnight at room temperature. Chitosan and alginate emulsions with oregano oil were made at a final concentration of 0.1% by dissolving 0.1 mL of oregano oil (W282812, Sigma Aldrich, Burlington, MA, USA) in 100 mL of the stocks of chitosan or alginate solutions and mixing until fully incorporated at room temperature. Alginate emulsion with olive oil was produced at a final concentration of 2% by dissolving 2 mL of olive oil (of extra virgin grade, procured bottled from a local convenience store) in 100 mL of the stock of alginate solution and mixing until well combined at room temperature. All stocks were finally sterilized at 121 °C for 15 min.

### 2.3. Application of the Edible Coatings and Their Emulsions to Meat Samples

Meat pieces were aseptically immersed in the edible coating solutions or their emulsions with essential oils for 1 min. The pieces that were immersed in alginate solutions were then submerged in calcium chloride solution (2% *w*/*v*) (CL00.0317, Chem-Lab, Zedelgem, Belgium), used as a crosslinking, for 30 s. All samples were dredged up and left in sterilized racks for 5 min to form a membrane and to drain off excess solution. Then, samples were packaged in polyamide/nylon/ polyethylene terephthalate barrier pouches (971055, Hendi B.V., De Klomp, The Netherlands) aerobically or under vacuum conditions by using a packaging machine (V.350, La.Va. Gmbh, Germany) and stored at 4 °C for 21 days in a Peltier cooled incubator (LIB-150M, Daihan Labtech Co Ltd., Gyeonggi-do, Republic of Korea). Eleven lots of samples were conducted: The first was the control, which consisted of meat samples without edible membrane stored aerobically. The second lot comprised of meat samples without edible membrane stored in vacuum packaging. The third and fourth lots were meat pieces with chitosan membrane stored aerobically or in vacuum packaging, respectively. The fifth and sixth lots consisted of meat samples with chitosan coating and oregano oil stored aerobically or in vacuum packaging, correspondingly. The seventh and eight lots comprised meat samples with alginate membrane stored aerobically or in vacuum packaging, respectively. The ninth and tenth lots were meat samples with alginate coating and oregano oil stored aerobically or in vacuum packaging, correspondingly. The final lot consisted of meat samples with alginate membrane and olive oil stored in vacuum packaging.

### 2.4. Microbiological Analysis

Total Mesophilic Counts (TMC), Total Psychrophilic Counts (TPC) and the populations of *Brochothrix thermosphacta* and lactic acid bacteria (LAB) were examined in meat samples, according to EN/ISO 4833-1:2013, EN/ISO 15214:1998 and EN/ISO 13722:2017, respectively, with modifications proposed by the Commission Regulation (EC) No. 2073/2005 on microbiological criteria for foodstuffs [25,26,27,28]. More specifically, 25 g of meat sample was transferred aseptically into a stomacher bag (Interscience, Saint Nom la Bretêche, France) containing 225 mL of sterile Maximum Recovery Diluent (MRD, CM0733 Oxoid, Basingstoke, UK) and homogenized in a stomacher (Lab Blender, Interscience) for 2 min. The appropriate serial decimal dilutions were also prepared in MRD solution. From each dilution, 0.1 mL of diluent was surface-inoculated in appropriate media. For the enumeration of TMC and TPC, the Plate Count Agar (PCA, 20500, Biolab, Budapest, Hungary) was used, whereas for the enumeration of *B. thermosphacta* and LAB counts the STAA and MRS agar (Biolab) were used, respectively. Incubation for TMC and LAB was performed at 37 °C for 48 h aerobically or in microaerophilic conditions, respectively, whereas incubation for TPC was performed aerobically at 7 °C for 7 d. STAA plates were incubated aerobically at 22 °C for 48 h. After incubation, the characteristic colonies were enumerated. Microbiological examination was performed before the application of edible coatings and on the 1st, 3rd, 7th, 14th and 21st day of storage.

### 2.5. Physicochemical Analyses

Meat samples were also examined for their colour and chemical composition. Meat colour was evaluated on freshly exposed meat samples immediately after unpacking, using the Konica Minolta CR-410 Chroma Meter with a 50 mm aperture size, illuminant C and a 2° observer. After calibration with a white tile (Y: 94.8/X: 0.3130/y: 0.3190), each meat sample was scanned three consecutive times at different positions, perpendicular to the myofibrils and avoiding fat and connective tissue. The measured values of lightness (L*), redness (a*) and yellowness (b*) were averaged over each sample. Moreover, the chroma and hue angle values of each sample were calculated based on the a* and b* averages according to the following formulae, as described by AMSA [29]:

$$chroma={\left(a^{*2}+b^{*2}\right)}^{1/2}$$

(1) $$h_{ab}=arctangent\;\left(b^{*}/a^{*}\right)$$

Regarding the chemical composition of meat pieces, 100 g of samples were comminuted with a Warring laboratory blender. The chemical analysis was performed in a near-infrared spectrometer (Perten DA7250, Perkin Elmer Ltd., Waltham, MA, USA) calibrated for meat and meat products measurement, in order to evaluate the moisture, total fats and total proteins of meat samples according to the manufacturer’s instructions. Physicochemical examination (colour, chemical composition) was performed before the application of edible coatings and on the 1st, 3rd, 7th, 14th and 21st day of storage.

### 2.6. Sensory Evaluation

Consumer evaluation was performed in the Laboratory of Animal Food Products Hygiene and Veterinary Public Health, School of Veterinary Medicine, in a place adequately adapted to perform a sensory evaluation. Sensory examination was performed on the 1st, 7th, 14th and 21st day of storage. Meat pieces, approximately 100 g each, were cooked on a pre-heated clam grill (Tefal Optigrill, Haute-Savoie, France) at 240 °C for 3 min at 200 °C until reaching an internal temperature of 72 °C. Each sample was cut into ten 2 × 2 cm cubes and kept warm (50 °C) until sensory evaluation, within 15 min after cooking. Ten experienced consumers were randomly selected among laboratory-staff and postgraduate students and were asked to evaluate taste, odour and tenderness of the cooked samples, using a scale ranging from 1 = dislike extremely to 10 = like extremely. Consumers were asked to rinse their mouth with water and eat unsalted bread before evaluating each sample. The product was defined as unacceptable after development of first off-odour or off-taste.

### 2.7. Statistical Analysis

Statistical evaluation of data involved the use of both parametric (mixed linear model with repeated measures ANOVA, where time and treatment were within and between factors, respectively) and non-parametric (Kruskal–Wallis H test, Pearson’s chi-square test) statistical methods. Measures of central tendency and dispersion were calculated for each variable in order to reveal the sample characteristics. All analyses were conducted using the statistical software program IBM SPSS Statistics (v.27.0., IBM Corporation, Armonk, NY, USA). Significance was set at *p*-value ≤ 0.05, unless otherwise noted.

## 3. Results

### 3.1. Microbiological Analysis

**Figure 1:**
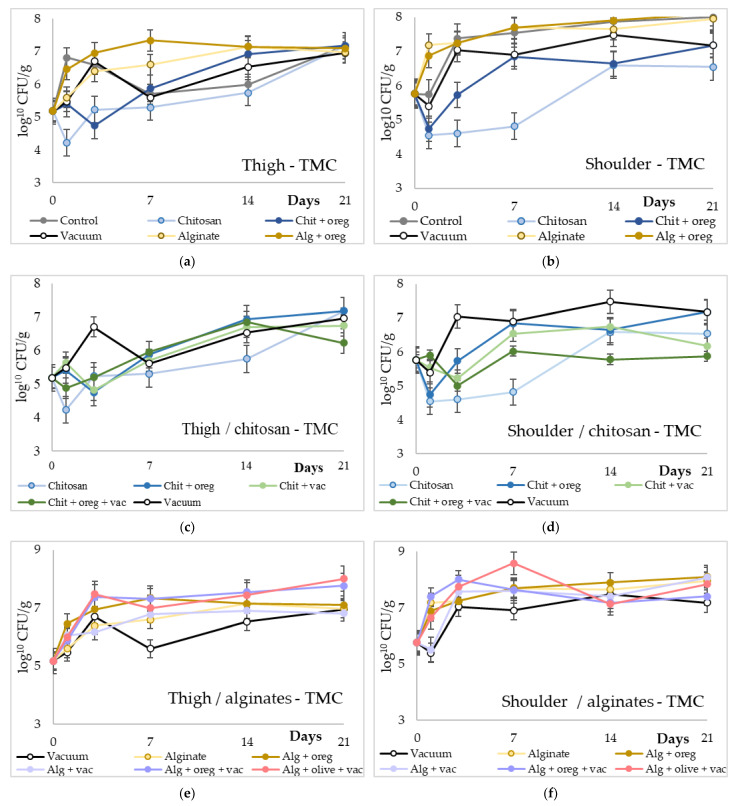
Total Mesophilic Counts (log10 cfu/g) of meat pieces over time (days). (**a**,**b**) TMC of control against chitosan or alginate coating with or without vacuum packaging in thigh and shoulder pieces; (**c**,**d**) TMC of different chitosan treatments in thigh and shoulder pieces; (**e**,**f**) TMC of different alginate treatments in thigh and shoulder pieces. (Chit: chitosan coating; oreg: oregano essential oil; vac: vacuum packaging; Alg: alginate coating; olive: olive oil addition).

**Figure 2:**
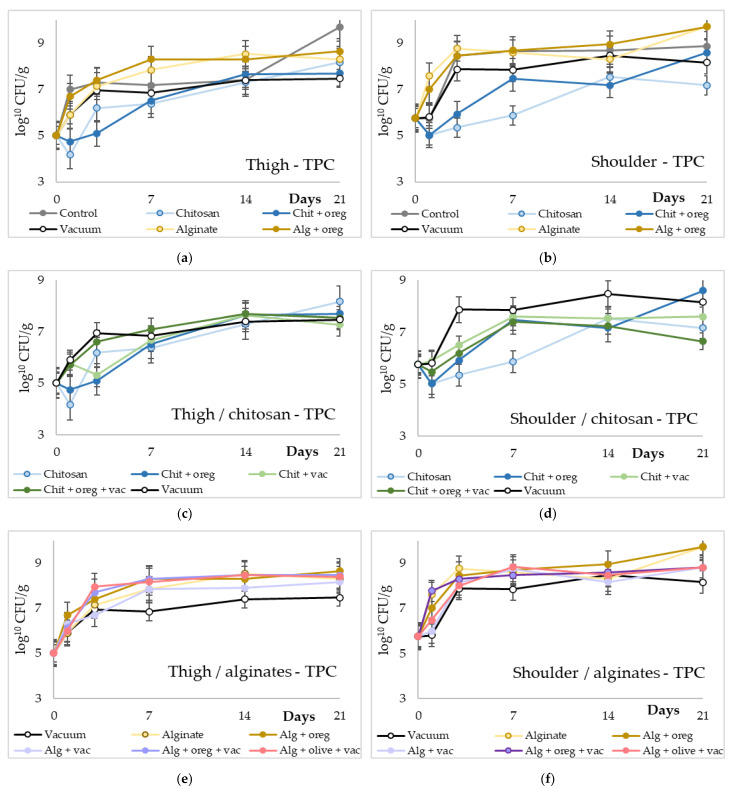
Total Psychrophilic Counts (log10 cfu/g) of meat pieces over time (days). (**a**,**b**) TPC of control lots against chitosan or alginate coating with or without vacuum packaging in thigh and shoulder pieces; (**c**,**d**) TPC of different chitosan treatments in thigh and shoulder pieces; (**e**,**f**) TPC of different alginate treatments in thigh and shoulder pieces. (Chit: chitosan coating; oreg: oregano essential oil; vac: vacuum packaging; Alg: alginate coating; olive: olive oil addition).

**Figure 3:**
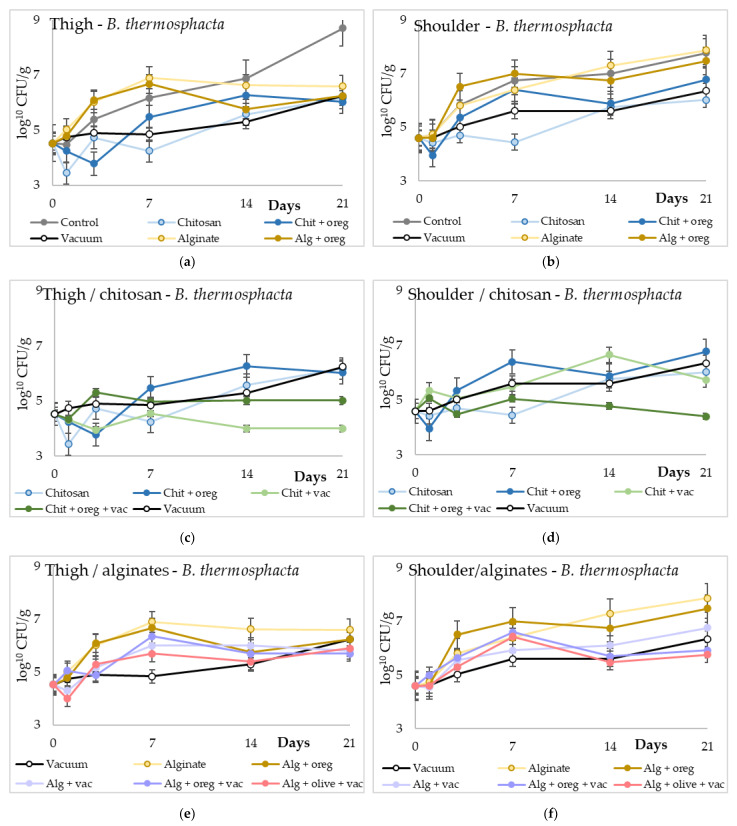
Populations of *B. thermosphacta* (log_10_ cfu/g) in meat pieces over time (days). (**a**,**b**) *B. thermosphacta* counts of control lots against chitosan or alginate coating with or without vacuum packaging in thigh and shoulder pieces; (**c**,**d**) *B. thermosphacta* counts of different chitosan treatments in thigh and shoulder pieces; (**e**,**f**) *B. thermosphacta* counts of different alginate treatments in thigh and shoulder pieces. (Chit: chitosan coating; oreg: oregano essential oil; vac: vacuum packaging; Alg: alginate coating; olive: olive oil addition).

**Figure 4:**
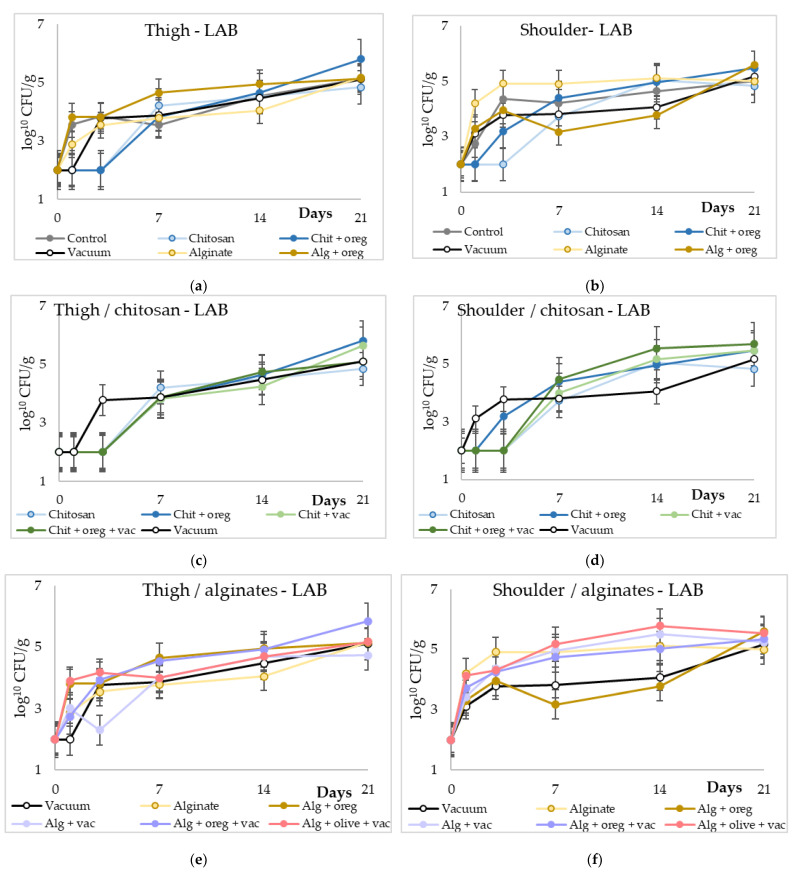
Populations of lactic acid bacteria (log_10_ cfu/g) in meat pieces over time (days). (**a**,**b**) LAB of control against chitosan or alginate coating with or without vacuum packaging in thigh and shoulder pieces; (**c**,**d**) LAB of different chitosan treatments in thigh and shoulder pieces; (**e**,**f**) LAB of different alginate treatments in thigh and shoulder pieces. (Chit: chitosan coating; oreg: oregano essential oil; vac: vacuum packaging; Alg: alginate coating; olive: olive oil addition).

The microbiological results are portrayed in Figure 1, Figure 2, Figure 3 and Figure 4 in an order suitable for comparison of the different treatments employed and reported in Supplementary Tables S1 and S2. Regarding the Total Mesophilic (TMC) and Psychrophilic (TPC) Counts, there was an increase in their population in all experimental groups over time. However, the population of TMC and TPC in chitosan lots (with and without oregano oil) was significantly lower (1–2 log_10_ cfu/g, *p*-value < 0.05) compared with the TMC and TPC of the control lot and the alginate lots (with and without oregano oil or olive oil). Moreover, the alginate lots had similar TMC and TPC with the control lot. The addition of oregano oil or olive oil in chitosan and alginate coatings did not affect their antimicrobial activity. Meat pieces from the shoulder region stored under vacuum had significantly higher TMC and TPC (*p*-value < 0.05) compared with the TMC and TPC of the chitosan lots. However, the TPC of vacuum-stored meat pieces from shoulder region was 1–2 log_10_ lower (*p*-value < 0.05) compared with the TPC of the alginate lots. No other significant differences were observed between the meat pieces from thigh region and meat pieces from shoulder region.

A similar trend was observed in the populations of *B. thermosphacta*. Over time, an increase in its population was observed in all experimental groups. Meat pieces with chitosan coating stored aerobically or under vacuum, as well as meat pieces coated with chitosan and oregano oil and stored under vacuum, had significantly lower (*p*-value < 0.05) populations compared with the control and alginate lots. The populations of *B. thermosphacta* of alginate lots were similar with the ones of the control lots. The incorporation of oregano or olive oil in the chitosan and alginate membranes did not influence the populations of *B. thermosphacta*. No significant differences in the populations were observed between the vacuum-stored lots and the rest of the experimental groups. Similar results were also observed between the thigh meat pieces and the shoulder meat pieces.

The population of lactic acid bacteria expressed a different trend compared with the other bacterial populations. No significant differences were observed between the control lots and the rest of the experimental groups. However, the chitosan lots had significantly lower LAB compared with the alginate lots. The initial population of LAB in meat pieces from both thigh and shoulder region was ~10^2^ CFU/g and their population increased 2–4 log_10_ cfu/g till the 21st day of the experiment. Vacuum storage and essential or vegetable oils did not alter the population of LAB.

### 3.2. Physicochemical Analyses

Descriptive statistics of all the parameters of meat colour are reported in Supplementary Tables S3 and S4, whereas the results concerning meat chemical composition are summarized in Table 1. Concerning meat colour, vacuum stored lots and most of the alginate lots had significantly lower lightness (L*, *p*-value < 0.05) compared with the one of chitosan lots. Decreased L* values were observed in meat pieces from both thigh and shoulder, especially in meat pieces stored under vacuum or treated with alginate and oregano oil and stored under vacuum. However, most of the chitosan lots had a lower redness value (a*) and chroma value (*p*-value < 0.05) compared with the vacuum stored lots and alginate lots. In fact, meat pieces from the shoulder region coated with alginate coatings and olive oil and stored under vacuum had significantly higher a* and chroma values (*p*-value < 0.05) compared with the other experimental groups. Still, there was a slight decrease in a* and chroma values in all experimental groups over time. The yellowness value (b*) and hue angle of most of the experimental groups did not differ from the values of the control lot. However, all alginate-coated meat pieces from the shoulder region that were stored under vacuum (plain alginate coatings, alginate emulsions with oregano or olive oil) had significantly higher b* value and hue angle (*p*-value < 0.05) compared with the ones of the rest of the lots. Still, meat pieces from the shoulder treated with alginate and oregano oil and stored aerobically had a significantly lower b* value and hue angle (*p*-value < 0.05), in comparison with the rest of the experimental groups.

Regarding the chemical composition of meat pieces, no significant differences were observed among the experimental groups. The moisture, total fats and total proteins of meat pieces from the thigh region ranged from 69.3–77.7%, 2.3–12.7% and 16–21.6%, respectively, whereas moisture, total fats and total proteins of meat pieces from the shoulder region ranged from 68.8–76.9%, 2.8–11.6% and 15.1–20%, correspondingly. The chemical composition of meat pieces did not alter over time. Table 1 presents the chemical characteristics of meat pieces.

### 3.3. Sensory Evaluation

**Figure 5:**
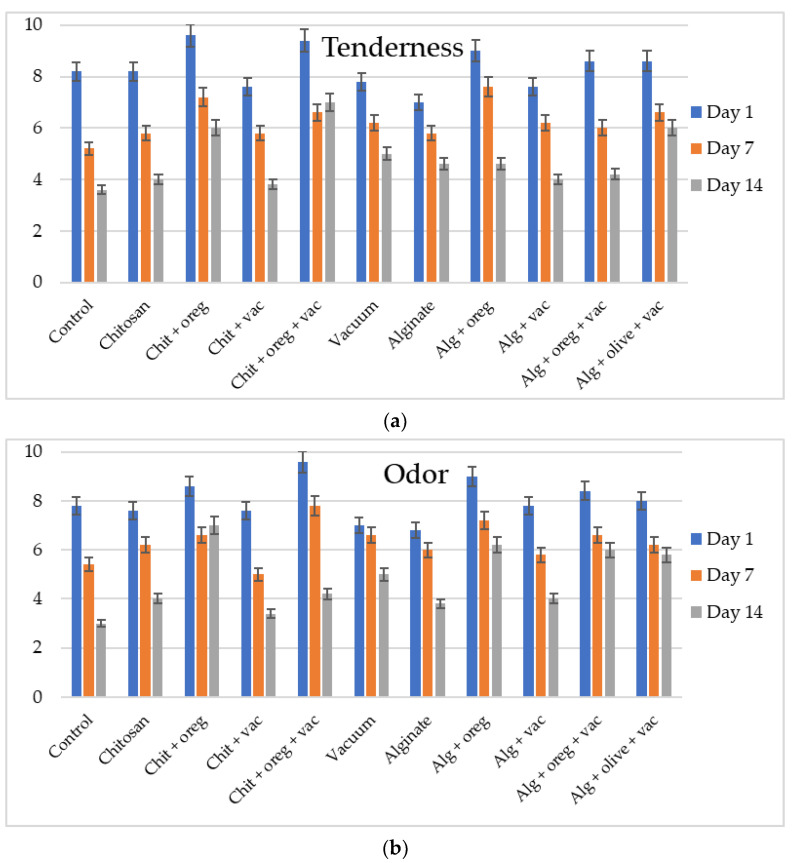

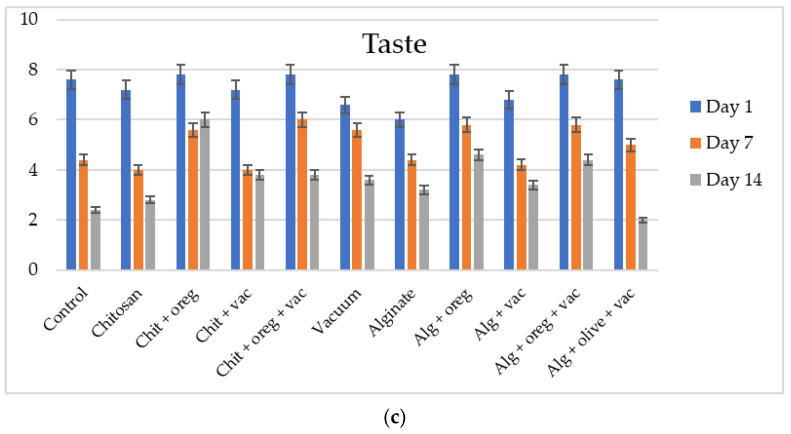
Results of sensory evaluation of meat pieces from the shoulder region over time. (Chit: chitosan coating; oreg: oregano essential oil; vac: vacuum packaging; Alg: alginate coating; olive: olive oil addition) ((**a**): tenderness; (**b**): odour; (**c**): taste).

Oregano oil positively affected the organoleptic properties of meat products. Meat pieces that were coated with chitosan or alginate coatings and oregano oil and stored either aerobically or under vacuum had significantly higher sensory scores (*p*-value < 0.05) regarding their taste, odour and tenderness. Meat pieces were considered as unacceptable for evaluation on the 21st day of storage, due to the development of off-odour. The results of sensory evaluation are illustrated in Figure 5.

## 4. Discussion

The objective of the study was to determine the effect of edible coatings, combined with oregano essential oil or olive oil, on the quality properties of mutton. To our knowledge, this is the first study comparing the effect of chitosan coatings and their emulsions with oregano and olive oil with the effect of alginate emulsions on the characteristics of mutton and on the extension of its shelf-life. For this reason, microbiological, physicochemical analysis and sensory evaluation of mutton, which was treated with edible coatings and essential or vegetable oils, were held at regular intervals during a 21-day storage period.

### 4.1. Microbiological Analysis

Chitosan coatings had a negative effect on the growth of TMC, TPC and the populations of *B. thermosphacta* and lactic-acid bacteria. This is in accordance with the results of Pabast et al. [30], who stated that chitosan membranes had slowed down the microbial growth of TMC, *Pseudomonas* spp. and LAB in lamb meat pieces, in comparison with the ones of the control samples. Chitosan coatings also reduced the TMC and the populations of *Pseudomonas* spp., *B. thermosphacta* and LAB in ground meat by 0.4–2.0 log_10_ cfu/g [31], whereas Kanatt et al. [32] mentioned that chitosan coatings of mutton kebabs reduced the TMC by up to 3 log_10_ cfu/g, compared with the uncoated ones. He et al. [33] claimed that chitosan coatings extended the shelf-life of mutton by up to three days. In our study, the TMC and TPC of the control lots exceeded 7 log_10_ cfu/g, which is considered the limit of spoilage level for meat [31], at 3 days of storage, whereas the TMC and TPC of chitosan-coated pieces reached the limit of 7 log_10_ cfu/g at 21 and 14 days of storage, respectively. The antimicrobial activity of chitosan has been attributed to three main mechanisms: the disruption of the cell membrane, due to the cationic properties of chitosan, the selective binding with metals that are viable for the microbial metabolism, due to its chelating characteristics, and the formation of an impermeable polymeric layer on the surface of cells that blocks the entry of nutrients and the excretion of toxic compounds [6].

On the other hand, the TMC, TPC and the populations of *B. thermosphacta* and LAB of alginate lots were similar with the ones of the control lots. This is consistent with the findings of Hamedi et al. [34], who stated that alginate coatings had no significant effect on decreasing the microbial load of TMC, TPC and LAB in chicken fillets. Similarly, the TMC, TPC and LAB of alginate-coated rainbow trout fillets had minor differences with the ones of the control samples [35]. However, other studies claimed that alginate membranes significantly reduced the TMC and TPC of food products such as buffalo meat patties and rainbow trout [36,37]. This can be attributed to the different composition of alginate coatings, namely the incorporation of sodium ascorbate and citric acid in them, the different type of meat and food packaging as well as the lower initial microbial populations of the food products, compared with this study.

In our study, the addition of oregano oil or olive oil in the edible coatings did not affect the growth of TMC, TPC, *B. thermosphacta* and LAB. Even though it has been stated that oregano oil possesses antimicrobial properties due to its high concentrations of carvacrol and thymol [38], according to our results, oregano oil did not show any synergistic effect with the edible coatings. Similarly, Vergara et al. [39] highlighted that lamb meat burgers, which were treated with oregano oil, had similar TVC, *Pseudomonas* spp. and LAB counts with the control burgers, with these microbial counts significantly increasing over time. Fernandes et al. [40] stated that the TMC of sheep burgers with 1000 ppm of oregano extract were similar with the control burgers throughout the storage period. However, LAB counts increased over time in all treatments and after 20 days of storage, the control burgers presented significantly higher LAB counts than the oregano-treated burgers. In another study, the TPC of lamb meat coated with oregano active coatings were approximately 2.0 log_10_ cfu/g lower than the controls, after the eighth day of storage [41]. Regarding olive coatings, Rubel et al. [42] mentioned that the TMC of mutton meatballs with 0.3% olive leaf extract were significantly lower in comparison with meatballs with no extract, whereas Martiny et al. [43] stated that coatings with olive leaf extract reduced the TPC of lamb meat fivefold. The different forms of oregano and olive oil, as well as the lower initial microbial counts of sheep meat and the different type of food packaging used in the other studies, could explain the differences of their results with the results of the current study.

The combined effect of edible coatings and vacuum packaging on the microbial counts of sheep meat was also investigated. Vacuum packaging did not influence the antimicrobial activity of chitosan or alginate, as no significant differences were observed in the microbial populations of meat pieces that were coated with an edible membrane and were stored either aerobically or under vacuum. This is in accordance with Assanti et al. [44], who stated that no significant differences were observed in the TMC and the populations of *B. thermosphacta* and LAB between chitosan-coated chicken burgers that were stored aerobically and the ones that were stored in vacuum packaging. The researchers attributed this observation to the greater antimicrobial activity of chitosan, compared with vacuum packaging. On the other hand, Duran and Kahve [45] suggested that the application of chitosan coatings and vacuum packaging was significantly more effective on the reduction of the proliferation rates of TMC and LAB, compared with the sole application of vacuum packaging. This difference to the results of the current study could be attributed to the different type of meat that was tested. Regarding sheep meat, Karabagias et al. [46] suggested the combination of thyme or oregano oil with modified atmosphere packaging (80% CO_2_/20% N_2_) for the greater antimicrobial activity and shelf-life extension of lamb meat.

### 4.2. Chemical Analysis

Regarding the colour characterization of sheep meat, lightness (L* value) is one of the primary colour parameters estimated, as it indicates the relative degree of black and white colour that is mixed with a given hue [29]. The alginate-coated meat pieces from the thigh region had lower L* values than the control pieces. This is in accordance with the findings of Vital et al. [47], who reported a decrease in the L* value of alginate-coated lamb patties compared with the control. The presence of exudates in the coated meat, as an effect of the edible coating, may explain the darkening of the meat colour. However, Guerrero et al. [48] stated that the addition of alginate coatings on lamb meat increased its lightness. Still, alginate lots and vacuum stored lots in the current study exhibited lower L* values compared with that of the chitosan lots, indicating the protective effect of alginate coatings and vacuum packaging on colour lightness. The darker colour of meat in vacuum packaging is associated with the formation of metmyoglobin, due to the absence of oxygen in the packaging bag [44,46]. Regarding chitosan coatings, Giatrakou et al. [49] claimed an increase in L* values in ready-to-eat chicken products with the incorporation of chitosan. In another study, the L* value of samples that contained chitosan 1% *w*/*v* showed a decreasing trend during storage [31]. The researchers attributed this observation to the water-holding capacity of chitosan which resulted in increased sample transparency and a lowering of lightness. However, the same study mentions that the L* value increased in samples packaged aerobically during storage, due to the action of bacterial proteases that results in the gradual protein decomposition and the increased light scattering on the protein macromolecules. This contradicts with the results of the current study, as the L* value was fluctuating and did not show an increasing or decreasing trend in the course of time, regardless of the type of packaging.

Redness (a* value) and chroma are colour parameters that are closely related to the freshness of meat. The alginate-coated meat pieces, with or without the incorporation of oregano and olive oil that were stored under vacuum, had higher a* value compared with the control pieces. This is consistent with the findings of Vital et al. [47], who stated that the treatment of lamb patties with alginate coatings and oregano oil resulted in higher a* values compared with the control patties. Guerrero et al. [48] claimed that, even though the addition of alginate coatings on lamb meat decreased its a* value after incorporation, the alginate-coated samples exhibited higher a* values and chroma during display compared with the control samples. Still, meat pieces from the shoulder treated with alginate and olive oil had higher a* values out of the rest of the experimental groups. Martiny et al. [43] mentioned that the a* value of lamb meat increased with the incorporation of olive leaf extract due to the phenolic compounds that are present in the extract. On the other hand, chitosan lots exhibited lower a* values in the current study compared with the one of vacuum stored lots and alginate lots. The lower a* value can be explained by the oxidation of myoglobin and the formation of metmyoglobin [41]. However, the a* value of chitosan-coated meat pieces was >10, indicating a bright red colour of meat. Park et al. [50] mentioned that the a* value of red meat increased as the amount of chitosan in the matrix increased, whereas Chounou et al. [31] claimed a higher a* value of chitosan-coated ground meat during storage, compared with the control. A slight decrease in a* and chroma values was observed over time in all experimental groups, which was greater in the control lots. The application of edible coating affects the alteration of meat colour during storage, as chitosan has a stabilizing effect on the red colour of meat [31] and alginate coatings slow down the oxygenation process in meat [48].

Concerning yellowness (b* value) and hue angle (colour tone) in general, no significant differences were observed among treatments. Vital et al. [47] highlighted an increase in the b* value in lamb patties, after the addition of alginate coatings and/or oregano oil, in contrast with Guerrero et al. [43] who reported a decrease in the b* value in lamb meat, after alginate incorporation. Park et al. [50] and Chounou et al. [31] claimed an increase in the b* value in chitosan-treated red and ground meat, as the amount of chitosan in the matrix increased, due to the natural yellowish colour of chitosan. Still, in the current study, the alginate-coated meat pieces from the shoulder region that were stored under vacuum (plain alginate coatings, alginate emulsions with oregano or olive oil) had a significantly higher b* value and hue angle, whereas the ones with alginate and oregano oil that were stored aerobically had a significantly lower b* value and hue angle, compared with the rest of the experimental groups. The increase in b* value may be attributed to the accumulation of metmyoglobin, which is more rapid at low O_2_ concentrations [46]. Fernandes et al. [51] and Barbosa et al. [52] mentioned an increase in the b* value and hue angle of sheep sausages and lamb burgers, respectively, that were treated with oregano extract, due to its characteristic coloration. Similarly, an increase in b* value has been observed in lamb meat with olive leaf extract, due to the phenolic compounds of the extract [43]. The b* value of the experimental groups in the current study was not significantly altered during storage, which is in accordance with the results of Vital et al. [47], implying that the incorporation of an edible coating in meat products may protect against b* discoloration.

The chemical composition of meat pieces was also evaluated throughout their storage. The moisture, protein and fat content of meat pieces did not differ among treatments or change in the course of time. Vital et al. [47] used lamb patties, in order to evaluate the effects of edible coatings with oregano oil on meat quality. The proximate composition of lamb meat was 69.95% moisture, 19.00% protein and 6.50% fat, which is in accordance with the results of our study. Govaris et al. [38] evaluated the antimicrobial effect of oregano oil and nisin in minced sheep meat. The chemical analysis of minced sheep meat showed a protein content of 19.8 ± 0.1%, fat 6.4 ± 0.1% and moisture 72.1 ± 0.4%. On the other hand, on an experiment where Fernandes et al. [40] estimated the effects of oregano extract on sheep meat quality, the researchers stated that the moisture of sheep burgers was 67.34 ± 0.21%, which is lower than the one mentioned in the current study. In another study [42], the crude protein content of mutton meatballs treated with 0.1–0.3% olive leaf extract was 23.34–24.13%, which is higher compared with this study. However, the crude proteins reduced during the 10-day storage of meat. The differences observed in the chemical composition of sheep meat could be attributed to the different breeds of sheep and the different cuts of sheep meat used in the studies.

### 4.3. Sensory Evaluation

In this study, meat pieces that were treated with oregano oil scored significantly higher than the rest of the experimental groups. This is consistent with the results of Govaris et al. [38], who reported that the odour, taste and overall acceptability of minced sheep meat treated with 0.6% oregano oil were significantly higher than the control. On the other hand, two studies of the same group [40,53] stated that oregano extract did not impair consumers’ organoleptic acceptance of lamb burgers. No significant differences in sensory acceptance were observed between burgers produced with synthetic antioxidants and burgers that contained oregano extract at a concentration of 24.01 mL/kg [53]. The inconsistent results could be attributed to subjectivity and different consumer preferences, origins and the dietary habits of the targeted population in each study [2]. Regarding the effect of edible coatings, no significant organoleptic changes were observed due to the application of alginate or chitosan coatings in meat samples. Similarly, Kanatt et al. [32] mentioned that the initial scores of the sensory attributes of mutton seekh kebabs were not affected by chitosan coatings. No statistical differences were observed in terms of flavour, tenderness and acceptability between alginate-coated and control lamb patties [47]. Still, He et al. [33] stated that the sensory properties of chitosan-coated chilled mutton obtained a significantly higher score than the control group. In either case, the application of edible coatings in sheep meat is generally acceptable by consumers and, depending on the targeted population, it may improve or leave the organoleptic scores unaffected.

The taste, odour and tenderness of meat pieces of all the experimental groups reduced significantly during the storage period. The control lots received unacceptable sensory scores (<5) at day 7, whereas the scores of chitosan and alginate lots that had oregano oil declined more slowly than the rest of the experimental groups, as they received unacceptable scores at day 14 of storage. Similarly, Camo et al. [41] stated that active coatings with oregano extended the fresh odour and colour of lamb from 8 to 13 days, compared with the control, whereas Fernandes et al. [40] claimed that the presence of oregano extract in sheep burgers prevented the loss of their organoleptic qualities up to 15 days of storage. Rubel et al. [42] mentioned that the colour, flavour and acceptability of mutton meatballs with olive leaf extract decreased significantly during the 10-day period of storage. In another study, chitosan-coated lamb meat received unacceptable scores at 13 days of storage, as opposed to the control meat, which had unacceptable scores at 10 days of storage [30]. He et al. [33] reported that chilled mutton with chitosan coating remained in the acceptable range up to 15 days during refrigeration, in contrast to the control samples that became unacceptable after 6 days of storage. All experimental groups in this study were unacceptable according to the sensory evaluation on the 21st day of storage, due to the development of off-odour. Changes in off-odour were consistent with the microbial results that indicate meat spoilage. The off-odour and off-flavour development is the result of the metabolism of *B. thermosphacta* and LAB in vacuum packaged meat and *Pseudomonas* spp. in aerobically packaged meat [46].

## 5. Conclusions

The results of microbiological, physicochemical analysis and sensory evaluation indicated that edible coatings, as well as their combinations with oregano essential oil or olive oil, could attribute to the retention of good quality characteristics and the extension of the shelf-life of mutton. Chitosan coatings were effective in controlling the microbial spoilage of mutton by suppressing microbial proliferation. Alginate coatings had an effect on the appearance of mutton pieces giving a deeper red colour. No significant differences were observed in the chemical composition of meat pieces among the experimental groups, nor it was altered in the course of time. Among the coatings examined, the ones that included oregano oil were the most agreeable according to their sensory attributes of mutton. Chitosan coatings and vacuum packaging did not show any synergistic effect. The combination of chitosan with oregano oil was proven to be the most efficient in the maintenance of the microbiological populations, the improvement of the organoleptic characteristics and the considerable extension of shelf-life of mutton.

## Figures and Tables

**Table 1:** Chemical composition of mutton pieces.

		Thigh			Shoulder		
Lot	Storage (Days)	Moisture (%)	Total Fats (%)	Total Proteins (%)	Moisture (%)	Total Fats (%)	Total Proteins (%)
Control	0	71.05	9.60	18.50	75.25	6.20	17.55
	1	75.80	2.80	20.80	71.50	8.40	18.60
	3	73.30	5.60	20.80	75.70	4.40	19.60
	7	74.10	4.80	19.00	71.50	9.50	19.10
	14	72.20	6.50	20.20	73.90	5.80	18.80
	21	73.00	6.30	19.60	72.40	6.80	19.30
Chitosan	0	71.05	9.60	18.50	75.25	6.20	17.55
	1	74.60	3.90	18.80	72.10	7.60	18.90
	3	76.00	3.60	18.60	72.70	6.10	19.70
	7	75.60	5.00	18.20	71.10	8.50	19.70
	14	74.90	4.60	18.50	68.80	11.10	17.70
	21	74.30	5.40	17.90	74.00	6.70	18.40
Chitosan + oregano	0	71.05	9.60	18.50	75.25	6.20	17.55
	1	73.90	8.10	17.60	71.50	8.70	19.10
	3	72.90	7.40	18.00	72.50	8.10	18.00
	7	73.30	7.40	18.40	73.50	7.50	17.90
	14	75.40	3.80	19.60	72.50	5.90	19.20
	21	75.00	6.10	17.80	74.60	5.50	18.70
Chitosan + vacuum	0	71.05	9.60	18.50	75.25	6.20	17.55
	1	73.60	9.10	17.40	74.20	6.00	19.50
	3	73.80	5.30	19.80	75.50	5.10	18.10
	7	74.70	5.90	18.60	73.10	7.60	18.30
	14	73.40	7.40	18.50	73.60	8.40	16.90
	21	73.40	6.80	18.70	72.10	9.10	18.10
Chitosan + oregano + vacuum	0	71.05	9.60	18.50	75.25	6.20	17.55
	1	73.10	6.60	19.80	71.90	9.60	18.60
	3	74.50	3.90	20.10	75.80	4.50	19.20
	7	72.70	6.30	19.10	74.10	6.00	18.00
	14	74.90	3.90	20.20	73.40	5.20	20.00
	21	74.90	3.80	19.30	71.30	6.80	20.00
Vacuum	0	71.05	9.60	18.50	75.25	6.20	17.55
	1	72.80	6.70	21.00	74.70	4.90	19.50
	3	70.50	10.20	17.60	73.50	5.70	18.00
	7	72.90	8.40	17.70	71.30	8.00	19.60
	14	73.80	4.90	20.80	72.30	8.30	18.10
	21	73.00	4.20	20.90	72.90	7.00	19.60
Alginate	0	71.05	9.60	18.50	75.25	6.20	17.55
	1	75.50	4.30	18.90	74.20	7.70	17.00
	3	75.80	2.30	21.60	75.20	7.50	16.20
	7	74.90	5.10	18.70	76.90	2.80	18.60
	14	76.50	4.80	17.70	74.10	6.60	18.00
	21	76.00	5.80	16.50	72.60	8.30	17.20
Alginate + oregano	0	71.05	9.60	18.50	75.2	6.20	17.55
	1	77.20	4.40	17.30	76.20	4.20	18.60
	3	74.50	5.60	19.40	73.20	8.80	17.00
	7	76.90	4.80	18.00	75.50	5.50	17.90
	14	76.00	2.70	19.40	74.00	7.20	17.60
	21	69.30	11.10	17.80	73.20	7.50	17.70
Alginate + vacuum	0	71.05	9.60	18.50	75.25	6.20	17.55
	1	75.00	5.90	17.70	73.60	8.80	17.60
	3	76.80	3.50	18.90	74.50	7.30	17.20
	7	74.70	6.80	18.00	75.00	8.10	15.70
	14	75.50	6.30	19.00	69.60	11.60	18.80
	21	77.30	3.00	18.50	72.80	8.60	16.60
Alginate + oregano + vacuum	0	71.05	9.60	18.50	75.25	6.20	17.55
	1	74.10	5.10	20.30	73.90	8.00	17.00
	3	75.70	5.30	18.60	76.20	4.70	17.50
	7	73.50	6.30	18.30	74.10	6.30	17.70
	14	73.60	7.70	17.60	74.30	7.50	16.90
	21	76.80	5.10	17.10	73.60	8.50	17.10
Alginate + olive + vacuum	0	71.05	9.60	18.50	75.25	6.20	17.55
	1	73.60	8.60	16.90	73.00	8.80	17.40
	3	77.70	4.30	16.70	74.40	9.20	15.20
	7	76.50	4.70	17.70	75.80	6.00	16.30
	14	70.70	12.70	16.00	74.50	7.00	17.40
	21	76.10	5.60	16.80	74.00	11.20	15.10

## Data Availability

Not applicable.

## Supplementary Materials

The following supporting information can be downloaded at: https://www.mdpi.com/article/10.3390/microorganisms11020507/s1. Supplementary Table S1: Mean populations (in log_10_ cfu/g) of Total Mesophilic Counts, Total Psychrophilic Counts, *Brochothrix thermosphacta* and Lactic Acid Bacteria in meat pieces from shoulder region (standard deviation in parenthesis). Supplementary Table S2: Mean populations (in log_10_ cfu/g) of Total Mesophilic Counts, Total Psychrophilic Counts, *Brochothrix thermosphacta* and Lactic Acid Bacteria in meat pieces from thigh region (standard deviation in parenthesis). Supplementary Table S3: Mean colour values of meat pieces from the shoulder meat pieces (standard deviation in parenthesis). Supplementary Table S4: Mean colour values of meat pieces from thigh region (standard deviation in parenthesis).

## References

[1] González N., Marquès M., Nadal M., Domingo J.L. (2020). Meat consumption: Which are the current global risks? A review of recent (2010–2020) evidences. Food Res. Int..

[2] Tsitsos A., Economou V., Arsenos G., Kalitsis T., Argyriadou A., Theodouridis A. (2021). Greek and European consumer behaviour towards beef, lamb and mutton meat safety and quality: A review. Int. J. Agric. Resour. Gov. Ecol..

[3] Pethick D.W., Hocquette J., Scollan N.D., Dunshea F.R. (2021). Review: Improving the nutritional, sensory and market value of meat products from sheep and cattle. Animal.

[4] Tsitsos A., Economou V., Chouliara E., Ambrosiadis I., Arsenos G. (2022). A comparative study on microbiological and chemical characteristics of small ruminant carcasses from abattoirs in Greece. Foods.

[5] Zhou G.H., Xu X.L., Liu Y. (2010). Preservation technologies for fresh meat—A review. Meat Sci..

[6] Song D.-H., Hoa V.B., Kim H.W., Khang S.M., Cho S.-H., Ham J.-S., Seol K.-H. (2021). Edible Films on Meat and Meat Products. Coatings.

[7] Kumar S., Mukherjee A., Dutta J. (2020). Chitosan based nanocomposite films and coatings: Emerging antimicrobial food packaging alternatives. Trends Food Sci. Technol..

[8] Yuan G., Chen X., Li D. (2016). Chitosan films and coatings containing essential oils: The antioxidant and antimicrobial activity, and application in food systems. Food Res. Int..

[9] Economou V., Tsitsos A., Theodouridis A., Ambrosiadis I., Arsenos G. (2022). Effects of chitosan coatings on controlling *Listeria monocytogenes* and methicillin-resistant *Staphylococcus aureus* in beef and mutton cuts. Appl. Sci..

[10] Campos C.A., Gerschenson L.N., Flores S.K. (2011). Development of edible films and coatings with antimicrobial activity. Food Bioprocess Technol..

[11] Kontominas M.G. (2020). Use of alginates as food packaging materials. Foods.

[12] Vital A.C.P., Guerrero A., Monteschio J.D.O., Valero M.V., Carvalho C.B., De Abreu Filho B.A., Madrona G.S., Do Prado I.N. (2016). Effect of edible and active coating (with rosemary and oregano essential oils) on beef characteristics and consumer acceptability. PLoS ONE.

[13] Şen F., Uzunsoy İ., Baştürk E., Kahraman M.V. (2017). Antimicrobial agent-free hybrid cationic starch/sodium alginate polyelectrolyte films for food packaging materials. Carbohydr. Polym..

[14] Heydari R., Bavandi S., Javadian S.R. (2015). Effect of sodium alginate coating enriched with horsemint (*Mentha longifolia*) essential oil on the quality of bighead carp fillets during storage at 4 °C. Food Sci. Nutr..

[15] Ruiz-Navajas Y., Viuda-Martos M., Sendra E., Perez-Alvarez J.A., Fernández-López J. (2013). In vitro antibacterial and antioxidant properties of chitosan edible films incorporated with *Thymus moroderi* or *Thymus piperella* essential oils. Food Control.

[16] Asensio C.M., Nepote V., Grosso N.R. (2011). Chemical stability of extra-virgin olive oil added with oregano essential oil. J. Food Sci..

[17] Rodriguez-Garcia I., Silva-Espinoza B.A., Ortega-Ramirez L.A., Leyva J.M., Siddiqui M.W., Cruz-Valenzuela M.R., Gonzalez-Aguilar G.A., Ayala-Zavala J.F. (2016). Oregano Essential Oil as an Antimicrobial and Antioxidant Additive in Food Products. Crit. Rev. Food Sci. Nutr..

[18] EFSA Panel on Food Additives and Nutrient Sources added to Food (ANS) (2010). Scientific Opinion on the use of oregano and lemon balm extracts as a food additive on request of the European Commission. EFSA J..

[19] Sedaghat Doost A., Stevens C.V., Claeys M., Van Der Meeren P. (2019). Fundamental Study on the salt tolerance of oregano essential oil-in-water nanoemulsions containing Tween 80. Langmuir.

[20] Sedaghat Doost A., Devlieghere F., Stevens C.V., Claeys M., Van der Meeren P. (2020). Self-assembly of Tween 80 micelles as nanocargos for oregano and trans-cinnamaldehyde plant-derived compounds. Food Chem..

[21] Beriain M.J., Gómez I., Petri E., Insausti K., Sarriés M.V. (2011). The effects of olive oil emulsified alginate on the physicochemical, sensory, microbial, and fatty acid profiles of low-salt, inulin-enriched sausages. Meat Sci..

[22] Parreidt T.S., Schott M., Schmid M., Müller K. (2018). Effect of presence and concentration of plasticizers, vegetable oils, and surfactants on the properties of sodium-alginate-based edible coatings. Int. J. Mol. Sci..

[23] Stark A.H., Madar Z. (2002). Olive oil as a functional food: Epidemiology and nutritional approaches. Nutr. Rev..

[24] Acevedo-Fani A., Salvia-Trujillo L., Rojas-Graü M.A., Martín-Belloso O. (2015). Edible films from essential-oil-loaded nanoemulsions: Physicochemical characterization and antimicrobial properties. Food Hydrocoll..

[25] European Commission (2005). Commission Regulation (EC) No 2073/2005 of 15 November 2005 on microbiological criteria for foodstuffs (Text with EEA relevance). OJEU.

[26] (1998). Microbiology of food and animal feeding stuffs—Horizontal method for the enumeration of mesophilic lactic acid bacteria—Colony-count technique at 30 °C.

[27] (2013). Microbiology of the food chain—Horizontal method for the enumeration of microorganisms—Part 1: Colony count at 30 °C by the pour plate technique.

[28] (2017). Microbiology of the food chain—Enumeration of *Brochothrix* spp.—Colony-count technique.

[29] American Meat Science Association (AMSA) (2012). AMSA Meat Color Measurement Guidelines.

[30] Pabast M., Shariatifar N., Beikzadeh S., Jahed G. (2018). Effects of chitosan coatings incorporating with free or nano-encapsulated *Satureja* plant essential oil on quality characteristics of lamb meat. Food Control.

[31] Chounou N., Chouliara E., Mexis S.F., Stavros K., Georgantelis D., Kontominas M.G. (2013). Shelf life extension of ground meat stored at 4 °C using chitosan and an oxygen absorber. Int. J. Food Sci. Technol..

[32] Kanatt S.R., Rao M.S., Chawla S.P., Sharma A. (2013). Effects of chitosan coating on shelf-life of ready-to-cook meat products during chilled storage. LWT.

[33] He L., Zou L., Yang Q., Xia J., Zhou K., Zhu Y., Han X., Pu B., Hu B., Deng W. (2016). Antimicrobial activities of nisin, tea polyphenols, and chitosan and their combinations in chilled mutton. J. Food Sci..

[34] Hamedi H., Kargozari M., Shotorbani P.M., Mogadam N.B., Fahimdanesh M. (2017). A novel bioactive edible coating based on sodium alginate and galbanum gum incorporated with essential oil of *Ziziphora persica*: The antioxidant and antimicrobial activity, and application in food model. Food Hydrocoll..

[35] Raeisi M., Hashemi M., Aminzare M., Ghorbani Bidkorpeh F., Ebrahimi M., Jannat B., Tepe B., Noori S.M.A. (2020). Effects of sodium alginate and chitosan coating combined with three different essential oils on microbial and chemical attributes of rainbow trout fillets. J. Aquat. Food Prod. Technol..

[36] Chidanandaiah Keshri R.C., Sanyal M.K. (2009). Effect of sodium alginate coating with preservatives on the quality of meat patties during refrigerated (4 ± 1C) storage. J. Muscle Foods.

[37] Hamzeh A., Rezaei M. (2012). The effects of sodium alginate on quality of rainbow trout (*Oncorhynchus mykiss*) fillets stored at 4 ± 2 °C. J. Aquat. Food Prod. Technol..

[38] Govaris A., Solomakos N., Pexara A., Chatzopoulou P.S. (2010). The antimicrobial effect of oregano essential oil, nisin and their combination against *Salmonella* Enteritidis in minced sheep meat during refrigerated storage. Int. J. Food Microbiol..

[39] Vergara H., Cózar A., Rubio N. (2020). Effect of adding of different forms of oregano (*Origanum vulgare*) on lamb meat burgers quality during the storage time. CYTA J. Food.

[40] Fernandes R.P.P., Trindade M.A., Lorenzo J.M., Munekata P.E.S., de Melo M.P. (2016). Effects of oregano extract on oxidative, microbiological and sensory stability of sheep burgers packed in modified atmosphere. Food Control.

[41] Camo J., Beltrán J.A., Roncalés P. (2008). Extension of the display life of lamb with an antioxidant active packaging. Meat Sci..

[42] Rubel S.A., Yu Z.N., Murshed H.M., Islam S.M.A., Sultana D., Rahman S.M.E., Wang J. (2021). Addition of olive (*Olea europaea*) leaf extract as a source of natural antioxidant in mutton meatball stored at refrigeration temperature. J. Food Sci. Technol..

[43] Martiny T.R., Raghavan V., de Moraes C.C., da Rosa G.S., Dotto G.L. (2020). Bio-based active packaging: Carrageenan film with olive leaf extract for lamb meat preservation. Foods.

[44] Assanti E., Karabagias V.K., Karabagias I.K., Badeka A., Kontominas M.G. (2021). Shelf life evaluation of fresh chicken burgers based on the combination of chitosan dip and vacuum packaging under refrigerated storage. J. Food Sci. Technol..

[45] Duran A., Kahve H.I. (2020). The effect of chitosan coating and vacuum packaging on the microbiological and chemical properties of beef. Meat Sci..

[46] Karabagias I., Badeka A., Kontominas M.G. (2011). Shelf life extension of lamb meat using thyme or oregano essential oils and modified atmosphere packaging. Meat Sci..

[47] Vital A.C.P., Guerrero A., Guarnido P., Cordeiro Severino I., Olleta J.L., Blasco M., Nunes do Prado I., Maggi F., Campo M.D.M. (2021). Effect of active-edible coating and essential oils on lamb patties oxidation during display. Foods.

[48] Guerrero A., Ferrero S., Barahona M., Boito B., Lisbinski E., Maggi F., Sañudo C. (2020). Effects of active edible coating based on thyme and garlic essential oils on lamb meat shelf life after long-term frozen storage. J. Sci. Food Agric..

[49] Giatrakou V., Ntzimani A., Savvaidis I.N. (2010). Effect of chitosan and thyme oil on a ready to cook chicken product. Food Microbiol..

[50] Park S., Marsh K.S., Dawson P. (2010). Application of chitosan-incorporated LDPE film to sliced fresh red meats for shelf life extension. Meat Sci..

[51] Fernandes R.P.P., Trindade M.A., Lorenzo J.M., de Melo M.P. (2018). Assessment of the stability of sheep sausages with the addition of different concentrations of *Origanum vulgare* extract during storage. Meat Sci..

[52] Barbosa T.C.M., Grisi C.V.B., da Fonseca S.B., Meireles B.R.L.D.A., Cordeiro A.M.T.D.M. (2022). Effect of active gelatin-starch film containing *Syzygium cumini* and *Origanum vulgare* extract on the preservation of lamb burgers. Meat Sci..

[53] Fernandes R.P.P., Trindade M.A., Tonin F.G., Pugine S.M.P., Lima C.G., Lorenzo J.M., de Melo M.P. (2017). Evaluation of oxidative stability of lamb burger with *Origanum vulgare* extract. Food Chem..

