# Supplementary material for: Effect of Chitosan and Alginate-Based Edible Membranes with Oregano Essential Oil and Olive Oil in the Microbiological, Physicochemical and Organoleptic Characteristics of Mutton
Source: Microorganisms. 2023 Feb 17;11(2):507. doi: 10.3390/microorganisms11020507 (PMC9961988; doi:10.3390/microorganisms11020507)
Supplement: Supplementary file 1 [file microorganisms-11-00507-s001.zip › microorganisms-2166678-supplementary/Supplementary Table S1.pdf]

Supplementary Table S1. Mean populations (in log_{10} cfu/gr) of Total Mesophilic Counts, Total Psychrophilic Counts, *Brochothrix thermosphacta* and Lactic Acid Bacteria in meat pieces from **shoulder region (standard deviation in parenthesis).**

Value	Storage (Days)	Treatment											Sig.
		Co	Ch	Ch + or	Ch + va	Ch + or + va	Va	Al	Al + or	Al + va	Al + or + va	Al + ol + va	
TMC	0	5.74 (0.19)^{Aa}	5.74 (0.19)^{Aab}	5.74 (0.19)^{Aab}	5.74 (0.19)^{Aabc}	5.74 (0.19)^{Aa}	5.74 (0.19)^{Aab}	5.74 (0.19)^{Aa}	5.74 (0.19)^{Aa}	5.74 (0.19)^{Aa}	5.74 (0.19)^{Aa}	5.74 (0.19)^{Aa}	NS
	1	5.72 (0.17)^{Aa}	4.50 (0.28)^{Aa}	4.50 (0.7)^{Aa}	5.53 (0.08)^{Aab}	5.88 (0.15)^{Aa}	5.30 (0.42)^{Aa}	6.58 (1.25)^{Aab}	6.08 (1.53)^{Aa}	5.43 (0.36)^{Aa}	7.30 (0.42)^{Aab}	6.40 (0.7)^{Aab}	NS
	3	7.25 (0.49)^{Cb}	4.58 (0.15)^{Aa}	5.45 (0.77)^{ABab}	5.03 (0.61)^{Aa}	5.00 (0.01)^{Aa}	6.80 (0.7)^{BCbc}	7.23 (0.08)^{Cab}	7.23 (0.08)^{Ca}	7.54 (0.21)^{Cb}	8.00 (0.01)^{Cb}	7.73 (0.05)^{Cbc}	***
	7	7.38 (0.55) ^{BCb}	4.81 (0.04) ^{Aa}	6.80 (0.28)^{BCb}	6.53 (0.08)^{ABCbc}	5.65 (0.91)^{ABa}	6.90 (0.01)^{BCbc}	7.66 (0.26) ^{Cab}	7.69 (0.12) ^{Ca}	7.60 (0.01)^{Cb}	7.40 (0.70)^{BCb}	8.34 (0.70)^{Cc}	***
	14	7.84 (0.21)^{CDb}	6.37 (0.66)^{ABb}	6.40 (0.70)^{ABCab}	6.72 (0.17)^{ABCDc}	5.65 (0.49) ^{Aa}	7.47 (0.01)^{BCDc}	7.65 (0.06)^{BCDab}	7.90 (0.01) ^{Da}	7.30 (0.42)^{BCDb}	7.15 (0.21)^{BCDab}	7.12 (0.06)^{BCDabc}	**
	21	8.00 (0.01)^{Db}	6.50 (0.28)^{ABCb}	7.15 (0.21)^{BCDb}	6.15 (0.21)^{ABabc}	5.84 (0.21)^{Aa}	7.15 (0.21)^{BCDbc}	7.95 (0.06)^{Db}	8.00 (0.42)^{Da}	8.06 (0.15)^{Db}	7.30 (0.42)^{CDab}	7.8 (0.28)^{Dbc}	***
Sig.		***	**	**	*	NS	***	***	NS	***	***	***	
TPC	0	5.75 (0.02) ^{Aa}	5.75 (0.02)^{Aabc}	5.75 (0.02)^{Aab}	5.75 (0.02)^{Aa}	5.75 (0.02) ^{Aa}	5.75 (0.02)^{Aa}	5.75 (0.02) ^{Aa}	5.75 (0.02) ^{Aa}	5.75 (0.02)^{Aa}	5.75 (0.02)^{Aa}	5.75 (0.02)^{Aa}	NS
	1	5.50 (0.70)^{ABa}	4.65 (0.91) ^{Aa}	4.65 (0.91)^{Aa}	5.88 (0.15)^{ABab}	5.34 (0.49)^{ABa}	5.73 (0.36)^{ABa}	6.84 (1.50)^{ABab}	6.50 (1.13)^{ABab}	5.93 (0.33)^{ABa}	7.75 (0.21)^{Bb}	6.45 (0.21)^{ABa}	*
	3	8.30 (0.56) ^{Cb}	5.32 (0.21) ^{Aab}	5.73 (0.61)^{Aab}	6.43 (0.36)^{ABabc}	5.82 (0.91)^{Aa}	7.87 (0.04)^{BCb}	8.77 (0.10)^{Cb}	8.30 (0.56)^{Cbc}	8.10 (0.28)^{BCb}	8.23 (0.33)^{Cbc}	8.00 (0.01)^{BCb}	***
	7	8.45 (0.63)^{BCb}	5.84 (0.21)^{Aabc}	7.34 (0.49)^{ABCbc}	7.53 (0.33)^{ABCc}	6.84 (1.20)^{ABa}	7.82 (0.18)^{BCb}	8.53 (0.33) ^{BCb}	8.66 (0.26)^{BCbc}	8.73 (0.05)^{BCb}	8.45 (0.21)^{BCbc}	8.80 (0.28)^{Cb}	**
	14	8.69 (0.12) ^{Cb}	7.38 (0.55)^{ABc}	7.15 (0.21) ^{Abc}	7.38 (0.55)^{ABbc}	7.19 (0.28) ^{Aa}	8.45 (0.21)^{BCb}	8.3 (0.01)^{BCab}	8.95 (0.06) ^{Cbc}	8.17 (0.01)^{ABCb}	8.6 (0.01)^{Cbc}	8.47 (0.01)^{Cb}	**
	21	8.84 (0.21)^{BCb}	7.15 (0.21)^{ABbc}	8.58 (0.15)^{BCc}	7.42 (0.59)^{ABbc}	6.65 (0.06) ^{Aa}	8.12 (0.24)^{ABCb}	9.50 (0.70) ^{Cb}	9.38 (0.86) ^{Cc}	8.73 (0.36)^{BCb}	8.73 (0.36)^{BCc}	8.73 (0.36)^{BCb}	**
Sig.		***	***	***	***	NS	***	***	***	***	***	***	

B. thermo-sphacta	0	4.55 (0.26)^{Aa}	4.55 (0.26)^{Aa}	4.55 (0.26)^{Aab}	4.55 (0.26)^{Aa}	4.55 (0.26)^{Aa}	4.55 (0.26)^{Aa}	4.55 (0.26)^{Aa}	4.55 (0.26)^{Aa}	4.55 (0.26)^{Aab}	4.55 (0.26)^{Aa}	4.55 (0.26)^{Aa}	NS
	1	4.50 (0.70)^{Aa}	4.00 (0.98)^{Aa}	3.95 (0.06)^{Aa}	5.15 (0.63)^{Aab}	5.03 (0.19)^{Aa}	4.42 (0.59)^{Aa}	4.50 (0.70)^{Aa}	4.58 (0.15)^{Aa}	4.50 (0.28)^{Aa}	4.99 (0.12)^{Aab}	4.53 (0.33)^{Aa}	NS
	3	5.77 (0.32)^{BCab}	4.60 (0.42)^{ABa}	5.27 (0.38)^{ABCab}	4.88 (0.58)^{ABab}	4.47 (0.01)^{Aa}	4.97 (0.28)^{ABa}	5.73 (0.36)^{ABCab}	6.50 (0.14)^{Cb}	5.50 (0.28)^{ABCabc}	5.62 (0.21)^{ABCbc}	5.30 (0.01)^{ABCab}	**
	7	6.50 (0.70)^{Cbc}	4.38 (0.30)^{Aa}	6.38 (0.12)^{BCb}	5.47 (0.10)^{ABCab}	4.80 (0.70)^{ABa}	5.60 (0.01)^{ABCab}	6.30 (0.42)^{BCbc}	7.00 (0.01)^{Cbc}	5.92 (0.10)^{ABCbc}	6.53 (0.33)^{Cd}	6.19 (0.70)^{BCb}	**
	14	7.00 (0.01)^{BCbc}	5.58 (0.58)^{ABa}	5.38 (1.11)^{ABab}	6.57 (0.38)^{BCb}	4.77 (0.10)^{Aa}	5.60 (0.01)^{ABab}	7.30 (0.01)^{Cbc}	6.69 (0.30)^{BCb}	6.03 (0.36)^{ABCc}	5.69 (0.12)^{ABCbc}	5.47 (0.01)^{ABab}	**
	21	7.77 (0.01)^{DEc}	5.60 (0.91)^{ABCa}	6.65 (0.49)^{BCDEb}	5.50 (0.70)^{ABab}	4.30 (0.42)^{Aa}	6.32 (0.21)^{BCDEb}	7.84 (0.21)^{Ec}	7.47 (0.01)^{CDEc}	6.50 (0.58)^{BCDEc}	5.90 (0.10)^{ABCDcd}	5.58 (0.58)^{ABCab}	***
**Sig.**		***	NS	***	**	NS	*	***	***	**	**	*	
**LAB**	0	2.00 (0.01)^{Aa}	2.00 (0.01)^{Aa}	2.00 (0.01)^{Aa}	2.00 (0.01)^{Aa}	2.00 (0.01)^{Aa}	2.00 (0.01)^{Aa}	2.00 (0.01)^{Aa}	2.00 (0.01)^{Aa}	2.00 (0.01)^{Aa}	2.00 (0.01)^{Aa}	2.00 (0.01)^{Aa}	NS
	1	2.50 (0.70)^{ABab}	2.00 (0.01)^{Aa}	2.00 (0.01)^{Aa}	2.00 (0.01)^{Aa}	2.00 (0.01)^{Aa}	2.69 (0.98)^{ABab}	4.19 (0.15)^{Bb}	3.23 (0.33)^{ABb}	3.08 (0.86)^{ABab}	3.30 (0.98)^{ABab}	4.12 (0.06)^{ABb}	*
	3	4.19 (0.58)^{BCabc}	2.00 (0.01)^{Aa}	2.70 (1.04)^{ABab}	2.00 (0.01)^{Aa}	2.00 (0.01)^{Aa}	3.65 (0.49)^{ABCabc}	4.67 (0.7)^{Cb}	3.95 (0.06)^{BCb}	4.32 (0.21)^{BCbc}	4.23 (0.08)^{BCbc}	4.23 (0.33)^{BCb}	**
	7	3.97 (0.7)^{Aabc}	3.45 (0.77)^{Aab}	4.38 (0.12)^{Abc}	3.38 (1.28)^{Aab}	3.53 (1.75)^{Aab}	3.8 (0.14)^{Aabc}	4.9 (0.01)^{Ab}	3.15 (0.21)^{Ab}	4.95 (0.06)^{Ac}	4.73 (0.05)^{Abc}	5.15 (0.21)^{Ab}	NS
	14	4.40 (0.70)^{ABCbc}	4.80 (0.70)^{ABCb}	4.86 (0.44)^{ABCc}	5.15 (0.21)^{ABCb}	5.38 (0.55)^{BCb}	4.05 (0.08)^{ABbc}	5.11 (0.01)^{ABCb}	3.77 (0.10)^{Ab}	5.40 (0.41)^{BCc}	5.02 (0.02)^{ABCc}	5.77 (0.10)^{Cb}	*
	21	5.00 (0.01)^{Ac}	4.77 (0.32)^{Ab}	5.45 (0.21)^{Ac}	5.34 (0.49)^{Ab}	5.69 (0.12)^{Ab}	5.15 (0.21)^{Ac}	5.00 (0.01)^{Ab}	5.53 (0.33)^{Ac}	5.08 (0.55)^{Ac}	5.32 (0.21)^{Ac}	4.57 (1.79)^{Ab}	NS
Sig.		***	***	***	***	***	***	***	***	***	***	***	

Treatment: Co: Control; Ch: Chitosan; Ch + or: Chitosan + oregano; Ch + va: Chitosan + vacuum; Ch + or + va: Chitosan + oregano + vacuum; Va: Vacuum; Al: Alginate; Al + or: Alginate + oregano; Al + va: Alginate + vacuum; Al + or + va: Alginate + oregano + vacuum; Al + ol + va: Alginate + olive + vacuum.

A-E Mean values in the same row (corresponding to the same days of storage) not followed by a common uppercase letter differ significantly (p-value < 0.05).

a-d Mean values in the same column (corresponding to the same treatment) not followed by a common lowercase letter differ significantly (p-value < 0.05).

* p-value < 0.05; ** p-value < 0.01; *** p-value < 0.001; NS no significance (p-value > 0.05)
