# Supplementary material for: Effect of Chitosan and Alginate-Based Edible Membranes with Oregano Essential Oil and Olive Oil in the Microbiological, Physicochemical and Organoleptic Characteristics of Mutton
Source: Microorganisms. 2023 Feb 17;11(2):507. doi: 10.3390/microorganisms11020507 (PMC9961988; doi:10.3390/microorganisms11020507)
Supplement: Supplementary file 1 [file microorganisms-11-00507-s001.zip › microorganisms-2166678-supplementary/Supplementary Table S2.pdf]

Supplementary Table S2. Mean populations (in log_{10} cfu/gr) of Total Mesophilic Counts, Total Psychrophilic Counts, *Brochothrix thermosphacta* and Lactic Acid Bacteria in meat pieces from thigh region (standard deviation in parenthesis).

Lot	Storage (Days)	Treatment											Sig.
		**Co**	**Ch**	**Ch + or**	**Ch + va**	**Ch + or + va**	**Va**	**Al**	**Al + or**	**Al + va**	**Al + or + va**	**Al + ol + va**	
**TMC**	0	5.18 *(0.20)*^{Aa}	5.18 *(0.2)*^{Aab}	5.18 *(0.20)*^{Aab}	5.18 *(0.20)* ^{Aab}	5.18 *(0.20)* ^{Aab}	5.18 *(0.20)* ^{Aa}	5.18 *(0.20)* ^{Aa}	5.18 *(0.20)*^{Aa}	5.18 *(0.20)* ^{Aa}	5.18 *(0.20)* ^{Aa}	5.18 *(0.20)* ^{Aa}	NS
	1	6.80 *(1.49)* ^{Aab}	4.21 *(0.70)* ^{Aa}	5.40 *(1.20)* ^{Aab}	5.63 *(0.79)* ^{Aab}	4.87 *(0.04)* ^{Aab}	5.47 *(0.01)* ^{Aab}	5.60 *(0.15)* ^{Aa}	6.46 *(0.56)*^{Aab}	6.06 *(0.19)* ^{Ab}	5.87 *(0.04)* ^{Aab}	6.00 *(0.01)* ^{Aab}	NS
	3	6.60 *(0.59)*^{ABCab}	5.23 *(0.61)*^{ABab}	4.74 *(0.30)* ^{Aa}	4.81 *(0.36)* ^{Aa}	5.19 *(1.04)* ^{Aa}	6.69 *(0.42)*^{ABCbc}	6.39 *(0.12)*^{ABCb}	6.95 *(0.06)*^{BCb}	6.17 *(0.21)*^{ABCb}	7.37 *(0.53)* ^{Cab}	7.47 *(0.49)* ^{Cbc}	*
	7	5.70 *(1.41)* ^{Aa}	5.30 *(0.33)* ^{Aab}	5.87 *(0.21)* ^{Aab}	5.96 *(0.12)* ^{Aab}	5.95 *(0.06)* ^{Aab}	5.60 *(0.01)* ^{Aab}	6.60 *(0.15)* ^{Abc}	7.34 *(0.70)*^{Ab}	6.77 *(0.01)* ^{Ac}	7.32 *(0.91)* ^{Aab}	7.00 *(0.01)* ^{Abc}	NS
	14	6.00 *(0.01)* ^{ABab}	5.74 *(0.17)* ^{Abc}	6.92 *(0.61)*^{ABab}	6.69 *(0.42)* ^{ABb}	6.84 *(0.08)* ^{ABb}	6.53 *(0.61)*^{ABabc}	7.14 *(0.28)* ^{ABc}	7.14 *(0.28)*^{ABb}	6.90 *(0.01)* ^{ABc}	7.54 *(0.55)* ^{Bb}	7.44 *(0.65)*^{ABbc}	*
	21	7.17 *(0.21)*^{ABb}	7.17 *(0.21)* ^{ABc}	7.17 *(0.21)* ^{ABb}	6.74 *(0.05)* ^{ABb}	6.21 *(0.70)*^{Aab}	6.95 *(0.06)* ^{ABc}	6.97 *(0.03)*^{ABbc}	7.09 *(0.42)*^{ABb}	6.81 *(0.14)* ^{ABc}	7.75 *(0.58)* ^{Bb}	8.00 *(0.01)* ^{Bc}	**
**Sig.**		***	***	***	***	*	***	***	***	***	***	***	
**TPC**	0	5.00 *(0.01)* ^{Aa}	5.00 *(0.01)* ^{Aab}	5.00 *(0.01)* ^{Aab}	5.00 *(0.01)* ^{Aa}	5.00 *(0.01)* ^{Aa}	5.00 *(0.01)* ^{Aa}	5.00 *(0.01)* ^{Aa}	5.00 *(0.01)*^{Aa}	5.00 *(0.01)* ^{Aa}	5.00 *(0.01)* ^{Aa}	5.00 *(0.01)* ^{Aa}	NS
	1	7.00 *(1.50)* ^{Aab}	4.17 *(0.21)* ^{Aa}	4.74 *(0.70)* ^{Aa}	5.77 *(0.49)* ^{Aab}	5.70 *(1.20)* ^{Aab}	5.90 *(0.15)* ^{Ab}	5.90 *(0.01)* ^{Ab}	6.69 *(0.67)*^{Ab}	6.30 *(0.15)* ^{Ab}	6.13 *(0.32)* ^{Ab}	6.00 *(0.01)* ^{Ab}	NS
	3	7.30 *(0.01)* ^{Cab}	6.19 *(0.91)*^{ABCabc}	5.09 *(0.42)*^{ABab}	5.31 *(1.00)* ^{Aa}	6.61 *(1.00)*^{ABCab}	6.95 *(0.06)*^{ABCcd}	7.14 *(0.28)* ^{BCc}	7.39 *(0.12)*^{Cbc}	6.69 *(0.01)* ^{ACb}	7.69 *(0.12)* ^{Cc}	7.95 *(0.06)* ^{Cc}	**
	7	7.17 *(0.21)* ^{ABab}	6.38 *(0.49)* ^{Abc}	6.51 *(0.04)* ^{Abc}	6.67 *(0.23)*^{Aabc}	7.09 *(0.12)* ^{ABab}	6.84 *(0.08)* ^{Ac}	7.84 *(0.08)* ^{BCd}	8.30 *(0.33)*^{Ccd}	7.84 *(0.28)* ^{BCc}	8.30 *(0.01)* ^{Ccd}	8.17 *(0.21)* ^{Ccd}	***
	14	7.39 *(0.42)*^{Aab}	7.30 *(0.33)* ^{Acd}	7.45 *(0.63)*^{ABCc}	7.65 *(0.06)*^{ABCDc}	7.69 *(0.12)*^{ABCDb}	7.39 *(0.12)*^{ABd}	8.54 *(0.08)* ^{De}	8.30 *(0.01)*^{ABCDcd}	7.90 *(0.15)*^{ABCDc}	8.45 *(0.21)*^{BCDd}	8.47 *(0.01)* ^{CDd}	*
	21	9.70 *(1.41)*^{Ab}	8.17 *(0.21)* ^{Ad}	7.69 *(0.42)* ^{Ac}	7.27 *(0.4)* ^{Abc}	7.54 *(0.28)* ^{Aab}	7.47 *(0.21)* ^{Ad}	8.3 *(0.01)*^{Ade}	8.65 *(0.06)*^{Ad}	8.17 *(0.21)* ^{Ac}	8.47 *(0.21)* ^{Ad}	8.39 *(0.21)* ^{Ad}	NS
**Sig.**		***	***	***	***	***	***	***	***	***	***	***	
B. thermosphacta	0	4.52 *(0.13)* ^{Aa}	4.52 *(0.13)* ^{Aab}	4.52 *(0.13)* ^{Aa}	4.52 *(0.13)* ^{Aa}	4.52 *(0.13)* ^{Aa}	4.52 *(0.13)* ^{Aa}	4.52 *(0.13)* ^{Aa}	4.52 *(0.13)*^{Aa}	4.52 *(0.13)* ^{Aab}	4.52 *(0.13)* ^{Aa}	4.52 *(0.13)* ^{Aa}	NS
	1	4.47 *(0.49)* ^{Aa}	3.43 *(0.30)* ^{Aa}	4.24 *(1.58)* ^{Aa}	4.32 *(0.91)* ^{Aa}	4.34 *(0.70)* ^{Aa}	4.74 *(0.05)* ^{Aa}	5.02 *(0.28)* ^{Aab}	4.77 *(0.49)*^{Aa}	4.30 *(0.33)* ^{Aa}	5.06 *(0.08)* ^{Aab}	4.00 *(0.01)* ^{Ab}	NS

	3	5.39 (*0.12*) ^{ABab}	4.72 (*0.81*) ^{ABab}	3.77 (*0.21*) ^{Aa}	3.95 (*0.06*) ^{ABa}	5.30 (*1.41*) ^{ABa}	4.90 (*0.15*) ^{ABa}	6.02 (*0.14*) ^{Bbc}	6.09 (*0.42*) ^{Bab}	5.17 (*0.21*) ^{ABbc}	4.87 (*0.04*) ^{ABab}	5.30 (*0.01*) ^{ABc}	*
	7	6.17 (*0.21*) ^{DEab}	4.24 (*0.28*) ^{Aab}	5.47 (*0.10*) ^{BCDa}	4.54 (*0.08*) ^{ABa}	4.97 (*0.40*) ^{ABCa}	4.84 (*0.08*) ^{ABa}	6.90 (*0.01*) ^{Ec}	6.67 (*0.51*) ^{DEb}	6.00 (*0.01*) ^{CDEd}	6.36 (*0.58*) ^{DEc}	5.69 (*0.12*) ^{CDcd}	***
	14	6.87 (*1.41*) ^{Aab}	5.57 (*0.12*) ^{Abc}	6.26 (*0.44*) ^{Aa}	4.00 (*0.01*) ^{Aa}	5.02 (*0.91*) ^{Aa}	5.30 (*0.01*) ^{Ab}	6.30 (*0.85*) ^{Abc}	5.75 (*0.58*) ^{Aab}	6.00 (*0.12*) ^{Ad}	5.69 (*0.01*) ^{Abc}	5.39 (*0.01*) ^{Ac}	NS
	21	8.71 (*1.07*) ^{Db}	6.17 (*0.21*) ^{BCc}	6.02 (*0.28*) ^{BCa}	4.00 (*0.01*) ^{Aa}	5.02 (*0.91*) ^{ABa}	6.24 (*0.08*) ^{BCc}	6.60 (*0.01*) ^{CDc}	6.24 (*0.55*) ^{BCab}	5.81 (*0.14*) ^{ABCcd}	5.69 (*0.12*) ^{ABCbc}	5.90 (*0.15*) ^{ABCd}	***
**Sig.**		***	***	***	NS	NS	**	***	***	***	***	**	
**LAB**	0	2.00 (*0.01*) ^{Aa}	2.00 (*0.01*) ^{Aa}	2.00 (*0.01*) ^{Aa}	2.00 (*0.01*) ^{Aa}	2.00 (*0.01*) ^{Aa}	2.00 (*0.01*) ^{Aa}	2.00 (*0.01*) ^{Aa}	2.00 (*0.01*) ^{Aa}	2.00 (*0.01*) ^{Aa}	2.00 (*0.01*) ^{Aa}	2.00 (*0.01*) ^{Aa}	NS
	1	3.55 (*0.49*) ^{ABb}	2.00 (*0.01*) ^{Aa}	2.00 (*0.01*) ^{Aa}	2.00 (*0.01*) ^{Aa}	2.00 (*0.01*) ^{Aa}	2.00 (*0.01*) ^{Aa}	2.87 (*0.04*) ^{ABb}	3.81 (*0.14*) ^{Bb}	3.02 (*0.91*) ^{ABabc}	2.74 (*0.70*) ^{ABa}	3.90 (*0.01*) ^{Bb}	*
	3	3.84 (*0.18*) ^{BCbc}	2.00 (*0.01*) ^{Aa}	2.00 (*0.01*) ^{Aa}	2.00 (*0.01*) ^{Aa}	2.00 (*0.01*) ^{Aa}	3.77 (*0.10*) ^{BCb}	3.54 (*0.28*) ^{Bc}	3.81 (*0.14*) ^{BCb}	2.23 (*0.33*) ^{Aab}	3.92 (*0.03*) ^{BCb}	4.17 (*0.01*) ^{Cb}	***
	7	3.54 (*0.08*) ^{Ab}	4.20 (*0.76*) ^{Ab}	3.81 (*0.36*) ^{Ab}	3.81 (*0.36*) ^{Ab}	3.87 (*0.21*) ^{Ab}	3.87 (*0.21*) ^{Ab}	3.77 (*0.21*) ^{Ac}	4.65 (*0.63*) ^{Abc}	4.00 (*0.01*) ^{Abcd}	4.54 (*0.28*) ^{Ab}	4.00 (*0.01*) ^{Ab}	NS
	14	4.54 (*0.08*) ^{Acd}	4.50 (*0.14*) ^{Ab}	4.65 (*0.63*) ^{Ab}	4.24 (*0.08*) ^{Ab}	4.74 (*0.05*) ^{Ac}	4.47 (*0.49*) ^{Abc}	4.04 (*0.11*) ^{Ac}	4.95 (*0.29*) ^{Abc}	4.69 (*0.01*) ^{Ad}	4.92 (*0.10*) ^{Abc}	4.69 (*0.01*) ^{Ac}	NS
	21	5.14 (*0.28*) ^{ABd}	4.84 (*0.28*) ^{ABb}	5.81 (*0.04*) ^{Bc}	5.65 (*0.06*) ^{ABc}	5.09 (*0.12*) ^{ABc}	5.11 (*0.36*) ^{ABc}	5.17 (*0.01*) ^{ABd}	5.14 (*0.28*) ^{ABc}	4.74 (*0.70*) ^{Acd}	5.84 (*0.08*) ^{Bc}	5.17 (*0.21*) ^{ABd}	*
Sig.		***	***	***	***	***	***	***	***	***	***	***	

Treatment: Co: Control; Ch: Chitosan; Ch + or: Chitosan + oregano; Ch + va: Chitosan + vacuum; Ch + or + va: Chitosan + oregano + vacuum; Va: Vacuum; Al: Alginate; Al + or: Alginate + oregano; Al + va: Alginate + vacuum; Al + or + va: Alginate + oregano + vacuum; Al + ol + va: Alginate + olive + vacuum.

$^{A-E}$ Mean values in the same row (corresponding to the same days of storage) not followed by a common uppercase letter differ significantly (p-value < 0.05).

$^{a-e}$ Mean values in the same column (corresponding to the same treatment) not followed by a common lowercase letter differ significantly (p-value < 0.05).

* p-value < 0.05; ** p-value < 0.01; *** p-value < 0.001; ^{NS} no significance (p-value > 0.05).
