# Supplementary material for: Effect of Chitosan and Alginate-Based Edible Membranes with Oregano Essential Oil and Olive Oil in the Microbiological, Physicochemical and Organoleptic Characteristics of Mutton
Source: Microorganisms. 2023 Feb 17;11(2):507. doi: 10.3390/microorganisms11020507 (PMC9961988; doi:10.3390/microorganisms11020507)
Supplement: Supplementary file 1 [file microorganisms-11-00507-s001.zip › microorganisms-2166678-supplementary/Supplementary Table S3.pdf]

microorganisms MDPI

Supplementary Table S3. Mean colour values of meat pieces from shoulder area(standard deviation in parenthesis).

Lot	Storage (Days)	Treatment											Sig.
		Co	Ch	Ch + Or	Ch + Va	Ch + Or + Va	Va	Al	Al + Or	Al + Va	Al + Or + Va	Al + Ol + Va	
L*	0	38.32 (3.52)^{Aa}	38.32 (3.52)^{Aa}	38.32 (3.52)^{Aa}	38.32 (3.52)^{Aa}	38.32 (3.52)^{Aa}	38.32 (3.52)^{Aa}	38.32 (3.52)^{Aab}	38.32 (3.52)^{Aa}	38.32 (3.52)^{Aa}	38.32 (3.52)^{Aa}	38.32 (3.52)^{Aa}	NS
	1	46.47 (0.19)^{BCc}	50.42 (0.14)^{CDd}	52.2 (4.85)^{DEb}	47.77 (0.38)^{BCDb}	56.1 (5.21)^{Ec}	43.93 (0.42)^{ABb}	40.87 (0.45)^{Abc}	50.98 (0.63)^{CDc}	43.47 (1.18)^{ABb}	49.64 (0.03)^{CDc}	46.76 (0.23)^{BCbc}	***
	3	40.14 (0.04)^{Aab}	49.19 (2.49)^{CDcd}	55.40 (0.25)^{Eb}	46.75 (0.85)^{BCb}	49.93 (5.74)^{CDbc}	42.78 (0.24)^{ABb}	41.66 (0.31)^{Abc}	41.31 (0.66)^{Aa}	52.77 (0.63)^{DEc}	44.01 (0.35)^{ABb}	49.03 (0.89)^{CDc}	***
	7	46.68 (0.12)^{EFc}	44.72 (0.48)^{CDEbc}	38.58 (2.37)^{Aa}	50.77 (0.76)^{Gb}	48 (0.29)^{Fbc}	43.18 (0.98)^{Cb}	43.92 (0.01)^{CDc}	45.30 (0.40)^{CDEb}	41.10 (1.32)^{Bab}	45.48 (0.12)^{DEb}	39.61 (0.19)^{ABa}	***
	14	43.6 (0.07)^{Cbc}	53.69 (1.42)^{Fd}	38.55 (0.72)^{Aa}	47.01 (1.20)^{Db}	52.22 (0.38)^{Ebc}	43.23 (0.18)^{Cb}	42.15 (0.19)^{Cc}	46.86 (0.40)^{Db}	40.29 (0.26)^{Bab}	42.86 (0.42)^{Cb}	39.58 (0.18)^{ABa}	***
	21	40.99 (0.16)^{BCab}	44.25 (0.17)^{EFb}	53.64 (0.01)^{Hb}	42.15 (1.49)^{CDa}	44.18 (0.53)^{EFab}	40.23 (0.84)^{Bab}	37.13 (0.40)^{Aa}	37.57 (0.57)^{Aa}	49.14 (1.14)^{Gc}	45.19 (0.55)^{Fb}	43.39 (0.12)^{DEb}	***
Sig.		***	***	***	***	***	*	***	***	***	*	***	
a*	0	21.54 (1.18)^{Ac}	21.54 (1.18)^{Aa}	21.54 (1.18)^{Ad}	21.54 (1.18)^{Aa}	21.54 (1.18)^{Ad}	21.54 (1.18)^{Abc}	21.54 (1.18)^{Ae}	21.54 (1.18)^{Ae}	21.54 (1.18)^{Ac}	21.54 (1.18)^{Abc}	21.54 (1.18)^{Ab}	NS
	1	21.10 (0.98)^{BCc}	11.57 (0.19)^{Ab}	32.30 (2.38)^{Ee}	22.79 (0.01)^{CDa}	24.64 (1.33)^{De}	20.78 (0.58)^{BCbc}	20.79 (0.60)^{BCde}	13.03 (0.21)^{Aa}	23.35 (0.62)^{Dd}	18.99 (0.37)^{Bab}	20.62 (0.40)^{BCb}	***
	3	16.60 (0.11)^{Ba}	14.51 (1.27)^{Ac}	13.88 (0.74)^{Ab}	17.74 (0.55)^{BCb}	12.94 (1.30)^{Ab}	19.82 (0.10)^{CDb}	19.53 (0.08)^{CDcd}	20.20 (0.24)^{Dd}	18.58 (0.31)^{BCDa}	18.58 (1.83)^{BCDa}	24.98 (1.18)^{Ec}	***
	7	17.92 (0.43)^{Ca}	14.8 (0.20)^{Ac}	16.65 (0.04)^{BCbc}	16.86 (1.88)^{BCb}	16.85 (0.1)^{BCc}	22.06 (1.03)^{Dc}	16.78 (0.36)^{BCb}	15.53 (0.03)^{ABbc}	20.46 (0.84)^{Dbc}	28.38 (0.12)^{Fd}	25.59 (0.26)^{Ecd}	***
	14	18.23 (0.91)^{Eab}	12.10 (0.53)^{Bb}	17.21 (0.34)^{Dc}	17.41 (0.72)^{DEb}	9.57 (0.05)^{Aa}	17.08 (0.01)^{Da}	15.18 (0.08)^{Ca}	16.69 (0.23)^{Dc}	23.59 (0.08)^{Fd}	22.94 (0.36)^{Fc}	26.88 (0.36)^{Gd}	***
	21	20.04 (0.14)^{EFbc}	18.49 (0.09)^{DEd}	9.57 (0.31)^{Aa}	16.92 (1.08)^{Cb}	16.01 (0.01)^{BCc}	20.31 (0.54)^{Fbc}	18.92 (0.17)^{DEFc}	15.21 (0.33)^{Bb}	19.60 (0.15)^{EFab}	16.51 (1.74)^{BCa}	17.49 (0.04)^{CDa}	***
Sig.		***	***	***	***	***	***	***	***	***	***	***	
b*	0	7.61 (0.20)^{Ac}	7.61 (0.20)^{Abc}	7.61 (0.20)^{Ab}	7.61 (0.20)^{Aa}	7.61 (0.20)^{Ac}	7.61 (0.20)^{Ac}	7.61 (0.20)^{Ad}	7.61 (0.20)^{Ad}	7.61 (0.20)^{Aa}	7.61 (0.20)^{Aa}	7.61 (0.20)^{Aa}	NS

	1	9.48 (*0.12*) ^{DEd}	8.23 (*0.09*) ^{BCDc}	14.73 (*1.56*) ^{Gc}	7.84 (*0.16*) ^{BCa}	12.07 (*0.77*) ^{Fd}	7.14 (*0.31*) ^{Bbc}	7.72 (*0.65*) ^{BCd}	3.90 (*0.14*) ^{Ab}	9.18 (*0.59*) ^{CDEb}	10.28 (*0.01*) ^{Eb}	9.43 (*0.41*) ^{DEb}	***
	3	3.16 (*0.18*) ^{Aa}	9.45 (*0.28*) ^{Ed}	5.06 (*1.17*) ^{Ba}	7.62 (*1.04*) ^{Da}	4.79 (*0.04*) ^{Ba}	5.88 (*0.16*) ^{BCb}	6.53 (*0.19*) ^{CDc}	7.28 (*0.43*) ^{Dd}	10.64 (*0.86*) ^{Ec}	10.23 (*0.09*) ^{Eb}	15.35 (*0.53*) ^{Fe}	***
	7	9.86 (*0.01*) ^{Fd}	6.17 (*0.02*) ^{Ba}	6.37 (*0.02*) ^{BCab}	7.60 (*0.98*) ^{DEa}	7.42 (*0.13*) ^{CDEbc}	6.43 (*0.87*) ^{BCbc}	6.74 (*0.19*) ^{BCDc}	4.77 (*0.08*) ^{Ac}	8.17 (*0.58*) ^{Eab}	13.63 (*0.24*) ^{Hc}	12.47 (*0.09*) ^{Gd}	***
	14	7.78 (*0.98*) ^{Cc}	7.28 (*0.45*) ^{Cb}	6.16 (*0.20*) ^{Bab}	7.81 (*0.28*) ^{Ca}	5.47 (*0.02*) ^{Ba}	3.80 (*0.92*) ^{Aa}	5.73 (*0.02*) ^{Bb}	4.42 (*0.21*) ^{Abc}	9.23 (*0.01*) ^{Db}	7.31 (*0.24*) ^{Ca}	10.8 (*0.29*) ^{Ebc}	***
	21	6.45 (*0.16*) ^{Cb}	5.96 (*0.19*) ^{Ca}	4.63 (*0.89*) ^{Ba}	4.42 (*0.11*) ^{Bb}	6.74 (*0.11*) ^{CDb}	6.55 (*0.72*) ^{Cbc}	4.72 (*0.19*) ^{Ba}	1.65 (*0.24*) ^{Aa}	8.19 (*0.70*) ^{Eab}	7.58 (*0.28*) ^{DEa}	8.34 (*0.16*) ^{Ea}	***
Sig.		***	***	***	***	***	***	***	***	***	***	***	
Chroma	0	22.85 (*1.05*) ^{Acd}	22.85 (*1.05*) ^{Aa}	22.85 (*1.05*) ^{Ad}	22.85 (*1.05*) ^{Aa}	22.85 (*1.05*) ^{Ad}	22.85 (*1.05*) ^{Ac}	22.85 (*1.05*) ^{Ad}	22.85 (*1.05*) ^{Ae}	22.85 (*1.05*) ^{Aa}	22.85 (*1.05*) ^{Abc}	22.85 (*1.05*) ^{Aa}	NS
	1	23.13 (*0.95*) ^{BCd}	14.2 (*0.09*) ^{Ab}	35.5 (*2.81*) ^{Ee}	24.1 (*0.05*) ^{BCa}	27.44 (*1.54*) ^{De}	21.97 (*0.65*) ^{Bbc}	22.18 (*0.79*) ^{Bd}	13.6 (*0.16*) ^{Aa}	25.09 (*0.79*) ^{CDb}	21.6 (*0.33*) ^{Bbc}	22.67 (*0.53*) ^{BCa}	***
	3	16.89 (*0.14*) ^{Ba}	17.32 (*1.22*) ^{BCc}	14.78 (*1.10*) ^{Ab}	19.31 (*0.91*) ^{CDb}	13.8 (*1.24*) ^{Ab}	20.67 (*0.14*) ^{Db}	20.59 (*0.01*) ^{Dc}	21.48 (*0.08*) ^{Dd}	21.41 (*0.69*) ^{Da}	21.22 (*1.64*) ^{Db}	29.32 (*1.28*) ^{Eb}	***
	7	20.45 (*0.38*) ^{Db}	16.04 (*0.18*) ^{Abc}	17.82 (*0.04*) ^{ABCbc}	18.49 (*2.12*) ^{Cb}	18.41 (*0.04*) ^{Cc}	22.98 (*1.23*) ^{Ec}	18.09 (*0.26*) ^{Bb}	16.25 (*0.05*) ^{ABbc}	22.03 (*0.99*) ^{DEa}	31.48 (*0.01*) ^{Gd}	28.47 (*0.27*) ^{Fb}	***
	14	19.82 (*1.22*) ^{Fb}	14.13 (*0.22*) ^{Bb}	18.28 (*0.39*) ^{Dc}	19.08 (*0.78*) ^{EFb}	11.02 (*0.03*) ^{Aa}	17.51 (*0.21*) ^{Da}	16.22 (*0.08*) ^{Ca}	17.27 (*0.28*) ^{CDc}	25.33 (*0.08*) ^{Hb}	24.08 (*0.41*) ^{Gc}	28.97 (*0.44*) ^{Ib}	***
	21	21.05 (*0.09*) ^{Ebc}	19.42 (*0.15*) ^{Dd}	10.64 (*0.67*) ^{Aa}	17.48 (*1.08*) ^{Cb}	17.37 (*0.05*) ^{Cc}	21.34 (*0.74*) ^{Ebc}	19.50 (*0.21*) ^{Dc}	15.30 (*0.30*) ^{Bb}	21.24 (*0.11*) ^{Ea}	18.18 (*1.46*) ^{CDa}	19.37 (*0.11*) ^{Dc}	***
Sig.		***	***	***	***	***	***	***	***	***	***	***	
Hue angle	0	0.34 (*0.02*) ^{Ab}	0.34 (*0.02*) ^{Aab}	0.34 (*0.02*) ^{Aa}	0.34 (*0.02*) ^{Ab}	0.34 (*0.02*) ^{Aa}	0.34 (*0.02*) ^{Ab}	0.34 (*0.02*) ^{Ab}	0.34 (*0.02*) ^{Acd}	0.34 (*0.02*) ^{Aa}	0.34 (*0.02*) ^{Aa}	0.34 (*0.02*) ^{Aa}	NS
	1	0.42 (*0.01*) ^{Dc}	0.61 (*0.01*) ^{Gd}	0.42 (*0.01*) ^{Dab}	0.33 (*0.01*) ^{Bb}	0.45 (*0.01*) ^{Ec}	0.33 (*0.01*) ^{Bb}	0.35 (*0.01*) ^{BCbc}	0.29 (*0.01*) ^{Ab}	0.37 (*0.01*) ^{Cab}	0.49 (*0.01*) ^{Fb}	0.42 (*0.01*) ^{Dc}	***
	3	0.18 (*0.01*) ^{Aa}	0.57 (*0.01*) ^{Ecd}	0.34 (*0.05*) ^{BCa}	0.40 (*0.03*) ^{Cc}	0.35 (*0.02*) ^{BCa}	0.28 (*0.01*) ^{Bab}	0.32 (*0.01*) ^{Bb}	0.34 (*0.02*) ^{BCd}	0.51 (*0.02*) ^{DEc}	0.50 (*0.03*) ^{Db}	0.55 (*0.01*) ^{DEd}	***
	7	0.50 (*0.01*) ^{Gd}	0.39 (*0.01*) ^{CDb}	0.36 (*0.01*) ^{Bab}	0.42 (*0.01*) ^{Ec}	0.41 (*0.01*) ^{DEbc}	0.28 (*0.02*) ^{Aab}	0.38 (*0.01*) ^{BCc}	0.29 (*0.01*) ^{Abc}	0.37 (*0.01*) ^{BCab}	0.44 (*0.01*) ^{Fb}	0.45 (*0.01*) ^{Fc}	***
	14	0.40 (*0.02*) ^{EFc}	0.54 (*0.04*) ^{Gc}	0.34 (*0.01*) ^{CDa}	0.42 (*0.01*) ^{Fc}	0.51 (*0.01*) ^{Gd}	0.21 (*0.05*) ^{Aa}	0.36 (*0.01*) ^{CDEbc}	0.25 (*0.01*) ^{ABb}	0.37 (*0.01*) ^{DEFab}	0.30 (*0.01*) ^{BCa}	0.38 (*0.01*) ^{DEFb}	***
	21	0.31 (*0.01*) ^{Cb}	0.31 (*0.01*) ^{Ca}	0.44 (*0.06*) ^{Db}	0.25 (*0.01*) ^{BCa}	0.39 (*0.01*) ^{Db}	0.30 (*0.02*) ^{Cb}	0.24 (*0.01*) ^{Ba}	0.10 (*0.01*) ^{Aa}	0.39 (*0.01*) ^{Db}	0.43 (*0.05*) ^{Db}	0.44 (*0.01*) ^{Dc}	***

Sig.	***	***	***	***	***	***	**	***	***	***	***

Treatment: Co: Control; Ch: Chitosan; Ch + Or: Chitosan + Oregano; Ch + Va: Chitosan + Vacuum; Ch + Or + Va: Chitosan + Oregano + Vacuum; Va: Vacuum; Al: Alginate; Al + Or: Alginate + Oregano; Al + Va: Alginate + Vacuum; Al + Or + Va: Alginate + Oregano + Vacuum; Al + Ol + Va: Alginate + Olive + Vacuum.

$^{A\text{-}H}$ Mean values in the same row (corresponding to the same days of storage) not followed by a common uppercase letter differ significantly (p-value < 0.05).

$^{a\text{-}e}$ Mean values in the same column (corresponding to the same treatment) not followed by a common lowercase letter differ significantly (p-value < 0.05).

* p-value < 0.05; ** p-value < 0.01; *** p-value < 0.001; ^{NS} no significance (p-value > 0.05)
