# Supplementary material for: Effect of Chitosan and Alginate-Based Edible Membranes with Oregano Essential Oil and Olive Oil in the Microbiological, Physicochemical and Organoleptic Characteristics of Mutton
Source: Microorganisms. 2023 Feb 17;11(2):507. doi: 10.3390/microorganisms11020507 (PMC9961988; doi:10.3390/microorganisms11020507)
Supplement: Supplementary file 1 [file microorganisms-11-00507-s001.zip › microorganisms-2166678-supplementary/Supplementary Table S4.pdf]

Supplementary Table S4. Mean colour values of meat pieces from thigh area (standard deviation in parenthesis).

Lot	Storage (Days)	Treatment											Sig.
		Co	Ch	Ch + Or	Ch + Va	Ch + Or + Va	Va	Al	Al + Or	Al + Va	Al + Or + Va	Al + Ol + Va	
L*	0	42.14 *(1.45)* ^{Ab}	42.14 *(1.45)* ^{Ab}	42.14 *(1.45)* ^{Ab}	42.14 *(1.45)* ^{Aa}	42.14 *(1.45)* ^{Ac}	42.14 *(1.45)* ^{Ac}	42.14 *(1.45)* ^{Ac}	42.14 *(1.45)* ^{Ac}	42.14 *(1.45)* ^{Ac}	42.14 *(1.45)* ^{Ab}	42.14 *(1.45)* ^{Abc}	NS
	1	47.40 *(2.40)* ^{EFc}	42.10 *(0.50)* ^{Dbc}	49.17 *(0.94)* ^{Fd}	46.70 *(0.02)* ^{Eb}	37.16 *(0.20)* ^{Aa}	38.10 *(0.70)* ^{ABb}	37.30 *(0.69)* ^{Aab}	40.80 *(0.45)* ^{CDbc}	40.03 *(0.03)* ^{BCb}	41.26 *(0.35)* ^{CDb}	40.46 *(0.12)* ^{CDb}	***
	3	37.77 *(0.38)* ^{CDa}	44.21 *(0.14)* ^{Fbc}	41.85 *(0.62)* ^{Eb}	49.18 *(0.86)* ^{Gb}	55.19 *(0.48)* ^{Hd}	38.28 *(1.23)* ^{Db}	35.08 *(0.89)* ^{Ba}	35.31 *(1.64)* ^{Ba}	33.15 *(0.30)* ^{Aa}	36.28 *(0.43)* ^{BCa}	43.23 *(0.25)* ^{EFbc}	***
	7	41.59 *(1.27)* ^{Cb}	44.25 *(0.88)* ^{DEc}	38.72 *(0.07)* ^{ABa}	46.32 *(0.14)* ^{Eb}	37.97 *(0.70)* ^{ABab}	40.51 *(0.54)* ^{BCbc}	37.10 *(1.40)* ^{Aab}	42.60 *(0.20)* ^{CDc}	38.50 *(0.01)* ^{ABb}	38.40 *(1.96)* ^{ABa}	49.18 *(1.85)* ^{Fd}	***
	14	46.29 *(0.11)* ^{Ec}	39.39 *(0.19)* ^{Ba}	38.48 *(0.31)* ^{ABa}	48.17 *(1.91)* ^{CDa}	36.47 *(0.13)* ^{Aa}	42.86 *(0.55)* ^{Dc}	51.72 *(1.95)* ^{Fd}	38.41 *(1.01)* ^{ABb}	40.37 *(1.05)* ^{BCbc}	43.46 *(0.04)* ^{Db}	43.35 *(1.13)* ^{Dc}	***
	21	41.70 *(0.06)* ^{FGb}	43.12 *(0.84)* ^{GHbc}	44.05 *(0.2)* ^{Hc}	40.43 *(1.65)* ^{DEFa}	39.88 *(1.65)* ^{DEFbc}	35.18 *(0.98)* ^{Aa}	38.69 *(0.07)* ^{CDb}	41.07 *(0.05)* ^{EFc}	39.29 *(0.59)* ^{CDEb}	37.68 *(0.57)* ^{BCa}	35.83 *(0.43)* ^{ABa}	***
Sig.		***	**	***	***	***	***	***	***	***	***	***	
a*	0	22.62 *(0.22)* ^{Ad}	22.62 *(0.22)* ^{Ac}	22.62 *(0.22)* ^{Af}	22.62 *(0.22)* ^{Ae}	22.62 *(0.22)* ^{Ae}	22.62 *(0.22)* ^{Ac}	22.62 *(0.22)* ^{Ad}	22.62 *(0.22)* ^{Ac}	22.62 *(0.22)* ^{Ac}	22.62 *(0.22)* ^{Ab}	22.62 *(0.22)* ^{Ac}	NS
	1	16.61 *(0.03)* ^{Aa}	20.17 *(0.73)* ^{Db}	17.00 *(0.45)* ^{Ac}	17.34 *(0.32)* ^{ABc}	18.83 *(0.11)* ^{Cc}	18.21 *(0.12)* ^{BCab}	22.8 *(0.02)* ^{Ed}	22.28 *(0.19)* ^{Ebc}	21.14 *(0.05)* ^{Db}	22.68 *(0.94)* ^{Eb}	17.33 *(0.56)* ^{ABa}	***
	3	20.66 *(0.41)* ^{DEFc}	21.22 *(0.36)* ^{EFb}	21.91 *(0.28)* ^{FGe}	25.92 *(0.03)* ^{Hf}	9.78 *(0.07)* ^{Aa}	18.46 *(0.67)* ^{BCab}	16.33 *(0.68)* ^{Ba}	18.75 *(2.78)* ^{CDab}	17.74 *(0.61)* ^{BCa}	23.73 *(0.18)* ^{Gb}	19.53 *(0.16)* ^{CDEab}	***
	7	16.72 *(0.38)* ^{Aa}	20.27 *(0.95)* ^{BCDEb}	18.17 *(0.24)* ^{ABd}	21.69 *(0.16)* ^{DEd}	18.60 *(0.42)* ^{ABCc}	19.53 *(0.21)* ^{BCDb}	20.61 *(0.77)* ^{CDEc}	18.02 *(1.38)* ^{ABa}	20.70 *(0.04)* ^{CDEb}	22.02 *(1.80)* ^{Eb}	21.05 *(2.02)* ^{CDEbc}	*
	14	19.75 *(0.22)* ^{Db}	13.43 *(0.21)* ^{Aa}	13.26 *(0.25)* ^{Ab}	16.46 *(0.19)* ^{Bb}	16.04 *(0.07)* ^{Bb}	22.21 *(0.25)* ^{EFc}	17.97 *(0.16)* ^{Cb}	23.58 *(1.83)* ^{Fc}	21.99 *(0.09)* ^{Ec}	22.12 *(0.31)* ^{EFb}	22.32 *(0.75)* ^{EFc}	***
	21	20.14 *(0.14)* ^{Ebc}	14.19 *(0.12)* ^{Ba}	12.15 *(0.01)* ^{Aa}	15.55 *(0.43)* ^{Ca}	21.68 *(0.25)* ^{Fd}	17.64 *(1.53)* ^{Da}	17.31 *(0.01)* ^{Dab}	15.05 *(0.06)* ^{Ca}	17.69 *(0.24)* ^{Da}	15.32 *(0.47)* ^{BCa}	17.43 *(0.02)* ^{Da}	***
Sig.		***	***	***	***	***	***	***	***	***	***	***	
b*	0	6.55 *(1.03)* ^{Aab}	6.55 *(1.03)* ^{Ab}	6.55 *(1.03)* ^{Ab}	6.55 *(1.03)* ^{Aa}	6.55 *(1.03)* ^{Ac}	6.55 *(1.03)* ^{Ab}	6.55 *(1.03)* ^{Ab}	6.55 *(1.03)* ^{Aa}	6.55 *(1.03)* ^{Aab}	6.55 *(1.03)* ^{Ab}	6.55 *(1.03)* ^{Ab}	NS

	**1**	5.80 *(0.01)*^{Ca}	4.91 *(0.09)*^{ABa}	6.72 *(0.16)*^{Db}	6.68 *(0.53)*^{Da}	5.33 *(0.01)*^{BCb}	4.58 *(0.09)*^{Aa}	10.28 *(0.30)*^{Gc}	9.03 *(0.12)*^{Fb}	11.20 *(0.13)*^{Hc}	7.75 *(0.19)*^{Eb}	5.77 *(0.54)*^{Cb}	***
	**3**	7.02 *(0.09)*^{CDEFb}	5.30 *(0.14)*^{ABCa}	8.34 *(0.25)*^{Fc}	7.77 *(2.62)*^{DEFa}	3.29 *(0.01)*^{Aa}	6.41 *(0.67)*^{BCDEFb}	4.21 *(0.22)*^{ABa}	5.95 *(1.44)*^{BCDEa}	5.54 *(0.28)*^{BCDa}	6.96 *(0.01)*^{CDEFb}	8.00 *(0.43)*^{EFc}	*
	**7**	5.67 *(0.26)*^{Ba}	6.50 *(0.05)*^{BCDb}	6.16 *(0.71)*^{BCb}	8.47 *(0.02)*^{Ea}	4.65 *(0.32)*^{Ab}	6.80 *(0.13)*^{CDb}	4.64 *(0.02)*^{Aa}	6.18 *(0.03)*^{CDa}	6.10 *(0.15)*^{BCab}	7.29 *(0.81)*^{Db}	11.93 *(0.26)*^{Fd}	***
	**14**	5.86 *(0.20)*^{Ba}	7.16 *(0.11)*^{Cb}	4.45 *(0.12)*^{Aa}	8.62 *(0.70)*^{Da}	4.49 *(0.15)*^{Ab}	4.49 *(0.30)*^{Aa}	9.11 *(0.72)*^{DEc}	6.05 *(0.19)*^{Ba}	7.38 *(0.59)*^{Cb}	9.65 *(0.14)*^{Ec}	8.59 *(0.43)*^{Dc}	***
	**21**	6.74 *(0.04)*^{Dab}	6.65 *(0.03)*^{Db}	7.50 *(0.09)*^{Dbc}	7.04 *(0.71)*^{Da}	8.94 *(0.20)*^{Ed}	3.17 *(1.11)*^{Ba}	4.12 *(0.19)*^{Ba}	6.67 *(0.15)*^{Da}	5.62 *(0.16)*^{Ca}	2.10 *(0.44)*^{Aa}	4.14 *(0.30)*^{Ba}	***
**Sig.**		*	***	***	***	***	***	***	***	***	***	***	
**Chroma**	**0**	23.56 *(0.49)*^{Ad}	23.56 *(0.49)*^{Ac}	23.56 *(0.49)*^{Ac}	23.56 *(0.49)*^{Ac}	23.56 *(0.49)*^{Ad}	23.56 *(0.49)*^{Ad}	23.56 *(0.49)*^{Ac}	23.56 *(0.49)*^{Ab}	23.56 *(0.49)*^{Ac}	23.56 *(0.49)*^{Ab}	23.56 *(0.49)*^{Ac}	NS
	**1**	17.59 *(0.02)*^{Aa}	20.76 *(0.73)*^{Db}	18.28 *(0.48)*^{ABb}	18.58 *(0.49)*^{ABCb}	19.56 *(0.11)*^{Cc}	18.78 *(0.09)*^{BCab}	25.01 *(0.1)*^{Ed}	24.04 *(0.22)*^{Eb}	23.92 *(0.01)*^{Ec}	23.96 *(0.96)*^{Eb}	18.26 *(0.70)*^{ABa}	***
	**3**	21.82 *(0.42)*^{DEc}	21.87 *(0.39)*^{DEb}	23.44 *(0.36)*^{EFc}	27.12 *(0.78)*^{Gd}	10.32 *(0.06)*^{Aa}	19.54 *(0.86)*^{CDab}	16.86 *(0.72)*^{Ba}	19.67 *(3.09)*^{CDa}	18.58 *(0.67)*^{BCa}	24.72 *(0.17)*^{Fb}	21.11 *(0.31)*^{DEb}	***
	**7**	17.65 *(0.44)*^{Aa}	21.29 *(0.89)*^{BCDb}	19.19 *(0.45)*^{ABb}	23.29 *(0.14)*^{DEc}	19.17 *(0.49)*^{ABc}	20.68 *(0.24)*^{BCbc}	21.12 *(0.75)*^{BCEb}	19.05 *(1.29)*^{ABa}	21.58 *(0.01)*^{CDb}	23.2 *(1.96)*^{DEb}	24.21 *(1.63)*^{Ec}	**
	**14**	20.60 *(0.27)*^{Db}	16.22 *(0.13)*^{ABa}	13.98 *(0.28)*^{Aa}	18.58 *(0.49)*^{Cb}	16.66 *(0.03)*^{Bb}	22.66 *(0.30)*^{Ecd}	20.15 *(0.47)*^{Db}	24.34 *(1.82)*^{Fb}	23.19 *(0.28)*^{EFc}	24.13 *(0.34)*^{EFb}	23.92 *(0.86)*^{EFc}	***
	**21**	21.23 *(0.14)*^{Fbc}	15.67 *(0.09)*^{BCa}	14.28 *(0.03)*^{Aa}	17.07 *(0.68)*^{Da}	23.45 *(0.31)*^{Gd}	17.93 *(1.70)*^{DEa}	17.79 *(0.03)*^{DEa}	16.87 *(0.01)*^{Ca}	18.56 *(0.27)*^{Ea}	15.47 *(0.40)*^{ABa}	17.91 *(0.09)*^{DEa}	***
**Sig.**		***	***	***	***	***	***	***	***	***	***	***	
**Hue angle**	**0**	0.28 *(0.03)*^{Aa}	0.28 *(0.03)*^{Aab}	0.28 *(0.03)*^{Aa}	0.28 *(0.03)*^{Aa}	0.28 *(0.03)*^{Aa}	0.28 *(0.03)*^{Acd}	0.28 *(0.03)*^{Ab}	0.28 *(0.03)*^{Aab}	0.28 *(0.03)*^{Aa}	0.28 *(0.03)*^{Ab}	0.28 *(0.03)*^{Aab}	NS
	**1**	0.33 *(0.01)*^{Cc}	0.23 *(0.01)*^{Aa}	0.37 *(0.01)*^{Db}	0.36 *(0.02)*^{Dab}	0.27 *(0.01)*^{Ba}	0.24 *(0.01)*^{Abc}	0.42 *(0.01)*^{Ec}	0.38 *(0.01)*^{Dcd}	0.48 *(0.01)*^{Fb}	0.32 *(0.01)*^{Cb}	0.32 *(0.01)*^{Cbc}	***
	**3**	0.32 *(0.01)*^{CDEbc}	0.24 *(0.01)*^{Aa}	0.36 *(0.01)*^{DEb}	0.29 *(0.09)*^{ABCDa}	0.32 *(0.01)*^{BCDEb}	0.33 *(0.02)*^{CDEd}	0.25 *(0.01)*^{ABab}	0.30 *(0.02)*^{ABCDab}	0.30 *(0.01)*^{ABCDa}	0.28 *(0.01)*^{ABCb}	0.38 *(0.01)*^{Ec}	*
	**7**	0.32 *(0.01)*^{CDbc}	0.31 *(0.01)*^{Cb}	0.32 *(0.03)*^{CDab}	0.37 *(0.01)*^{Dab}	0.24 *(0.01)*^{ABa}	0.33 *(0.01)*^{CDd}	0.22 *(0.01)*^{Aa}	0.33 *(0.02)*^{CDbc}	0.28 *(0.01)*^{BCa}	0.31 *(0.01)*^{Cb}	0.51 *(0.05)*^{Ed}	***
	**14**	0.28 *(0.01)*^{CDab}	0.48 *(0.01)*^{Gd}	0.32 *(0.01)*^{Dab}	0.48 *(0.02)*^{Gb}	0.27 *(0.01)*^{BCa}	0.19 *(0.01)*^{Aab}	0.46 *(0.02)*^{Gc}	0.25 *(0.01)*^{Ba}	0.32 *(0.02)*^{Da}	0.41 *(0.01)*^{Fc}	0.36 *(0.01)*^{Ec}	***
	**21**	0.32 *(0.01)*^{Cabc}	0.43 *(0.01)*^{Dc}	0.55 *(0.01)*^{Ec}	0.42 *(0.02)*^{Db}	0.39 *(0.01)*^{Dc}	0.17 *(0.04)*^{Aa}	0.23 *(0.01)*^{Bab}	0.40 *(0.01)*^{Dd}	0.30 *(0.01)*^{Ca}	0.13 *(0.03)*^{Aa}	0.23 *(0.01)*^{Ba}	***

**Sig.**	***	***	***	***	***	***	***	***	***	***	***

Treatment: Co: Control; Ch: Chitosan; Ch + Or: Chitosan + Oregano; Ch + Va: Chitosan + Vacuum; Ch + Or + Va: Chitosan + Oregano + Vacuum; Va: Vacuum; Al: Alginate; Al + Or: Alginate + Oregano; Al + Va: Alginate + Vacuum; Al + Or + Va: Alginate + Oregano + Vacuum; Al + Ol + Va: Alginate + Olive + Vacuum.
A-H Mean values in the same row (corresponding to the same days of storage) not followed by a common uppercase letter differ significantly (p-value < 0.05).
a-f Mean values in the same column (corresponding to the same treatment) not followed by a common lowercase letter differ significantly (p-value < 0.05).
* p-value < 0.05; ** p-value < 0.01; *** p-value < 0.001; NS no significance (p-value > 0.05
